# An Engineered *Escherichia coli* Strain with Synthetic Metabolism for in‐Cell Production of Translationally Active Methionine Derivatives

**DOI:** 10.1002/cbic.202000257

**Published:** 2020-10-13

**Authors:** Christian Johannes Schipp, Ying Ma, Ammar Al‐Shameri, Federico D'Alessio, Peter Neubauer, Roberto Contestabile, Nediljko Budisa, Martino Luigi di Salvo

**Affiliations:** ^1^ Chair of Bioprocess Engineering, Institute of Biotechnology Technische Universität Berlin ACK 24 Ackerstraße 76 13355 Berlin Germany; ^2^ Paraxel International GmbH, Berlin, Campus DRK Kliniken Berlin Westend Haus 18 Spandauer Damm 130 14050 Berlin Germany; ^3^ Institut für Chemie Technische Universität Berlin Müller-Breslau-Straße. 10 10623 Berlin Germany; ^4^ Dipartimento di Scienze Biochimiche “A. Rossi Fanelli” Sapienza Università di Roma Piazzale Aldo Moro, 5 – Edificio CU20 00185 Roma Italy; ^5^ Department of Chemistry University of Manitoba Winnipeg MB, R3T 2N2 Canada

**Keywords:** click chemistry, genetic code expansion, green chemistry, metabolic engineering, noncanonical amino acids, trans-sulfuration.

## Abstract

In the last decades, it has become clear that the canonical amino acid repertoire codified by the universal genetic code is not up to the needs of emerging biotechnologies. For this reason, extensive genetic code re‐engineering is essential to expand the scope of ribosomal protein translation, leading to reprogrammed microbial cells equipped with an alternative biochemical alphabet to be exploited as potential factories for biotechnological purposes. The prerequisite for this to happen is a continuous intracellular supply of noncanonical amino acids through synthetic metabolism from simple and cheap precursors. We have engineered an *Escherichia coli* bacterial system that fulfills these requirements through reconfiguration of the methionine biosynthetic pathway and the introduction of an exogenous direct trans‐sulfuration pathway. Our metabolic scheme operates *in vivo*, rescuing intermediates from core cell metabolism and combining them with small bio‐orthogonal compounds. Our reprogrammed *E. coli* strain is capable of the in‐cell production of l‐azidohomoalanine, which is directly incorporated into proteins in response to methionine codons. We thereby constructed a prototype suitable for economic, versatile, green sustainable chemistry, pushing towards enzyme chemistry and biotechnology‐based production.

## Graphical abstract

**Doing things differently**: We have metabolically engineered an *E. coli* strain by reconfiguring the methionine biosynthetic pathway to create a bacterial system with a trans‐sulfuration pathway for in‐cell production and incorporation of l‐azidohomoalanine. Our system is focused on green, sustainable chemistry, and only requires water, salts, trace elements, and simple carbon sources, pushing towards enzyme chemistry and biotechnology‐based production.

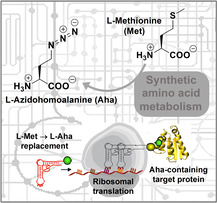

## Introduction

Xenobiology is an innovative discipline in engineering biology that focuses on the design of biological systems endowed with unusual biochemical features provided by chemical compounds of mostly anthropogenic origin.[^1^, ^2^] The evidence that life on Earth uses very few chemical compounds strongly contrasts with the exceptionally rich and wide repertoire of building blocks synthetized by organic chemists over the past 200 years.^[3]^ Therefore, in the 21st century, engineering biology will be able to bring potential that transcends traditional biotechnology, gene technologies, and even synthetic biology itself.^[4]^ In this perspective, the use of diverse synthetic building blocks to create artificial life is highly relevant for the new upcoming technologies. However, the key point on the path following this vision is the generation of self‐sustainable engineered cells able to operate with new sets of synthetic – mainly anthropogenic – metabolites, polymers and structures endowed by unusual biochemical features, novel energy sources, and alternative genetic codes.^[5]^ The simplest attempt to change the genetic code is to replace one canonical amino acid with its suitable noncanonical amino acid (ncAA) counterpart.^[6]^ Moreover, the amino acid repertoire codified by the standard genetic code can be either reduced or expanded.^[1]^ So far, most of the ncAAs replaced by genetic code reassignment have been chemically synthetized. However, the first step towards the creation of a synthetic life that operates with a truly alternative genetic code would be the self‐directed intracellular metabolic synthesis of the desired ncAAs.^[7]^ Synthetic cells with such capacity are *conditio sine qua non* for engineering self‐sustainable artificial life. Indeed, the incorporation of natural or synthetic ncAAs into proteins during translation is a very attractive approach to expand the scope of ribosomal protein synthesis beyond the 20 canonical amino acids, thus generating novel structural and functional protein diversity. In nature, the main source of chemical variety in mature proteins and peptides is achieved by post‐translational modifications (PTMs), as only a few polypeptide structures merely derive from the translation of their genes.^[8]^ On the other hand, the number of constituent amino acids in ribosome‐mediated protein synthesis is restricted to the 20 canonical amino acids; thus, nature uses PTMs to expand functional diversity. Mimicking these PTM machineries is experimentally not trivial and is particularly difficult for the production of large quantities of homogeneous proteins modified in a specific manner.^[9]^ The most straightforward way to solve this problem would be to insert the natural or synthetic ncAAs of interest directly during translation: in other words, to re‐engineer the genetic code.^[6]^ This methodology exploits the biological, chemical, and physical properties of the chosen amino acids that can be accurately defined by the chemist at the bench. In addition, taking advantage of the genetic encoding of these ncAAs, their ribosomal incorporation into peptides would occur with exquisite specificity; among the diverse opportunities offered by this methodology, a remarkable example is the incorporation of ncAAs to achieve enhanced chemical diversification of recombinantly produced antimicrobial peptides.[^10^, ^11^, ^12^]

Through the insertion of specific ncAAs, many opportunities for chemical coupling can be exploited; of particular interest is the use of ncAAs with bio‐orthogonal chemical functionalities such as azides, olefins, carbonyl compounds (ketones and aldehydes), strained and unstrained alkynes, halogens, oximes, hydrazones, boronic esters and acids, which can be easily and efficiently linked to a variety of ligands.^[13]^ Such bio‐orthogonal derivatizations, including efficient conjugations with sugars, other peptides, poly(ethylene glycol)s, optical markers and so forth,^[14]^ endow proteins with novel and unique functions, leading to improved structural stability, specificity, bioavailability and half‐life.[^15^, ^16^] Among the bio‐orthogonal chemistries developed for specific chemoselective modifications, the copper(I)‐catalyzed Huisgen cycloaddition reaction between azides and alkynes (also known as “click chemistry”) has found widespread application since its mild conditions allow full retention of the protein structure.[^17^, ^18^] This chemistry requires production of recombinant proteins with site‐ or residue‐specific incorporation of alkyne‐ or azide‐containing amino acid analogues by insertion of suitable ncAAs; proteins labeled in this way can be used for reactions with ligands containing complementary azide or alkyne derivatives. The incorporation of ncAAs typically requires the addition of the amino acid analogue in the growing medium, which is taken up by the cell machinery and used in protein translation.[^19^, ^20^] This approach, which implies a time‐consuming, sometimes expensive synthesis of the amino acid analogue, is feasible for small‐scale experiments, but might not be adequate for large‐scale production. Also, it may be limited by the substrate promiscuity of the native amino acid transporters in the cell.

Herein, we present an innovative, preliminary semisynthetic method which circumvents these difficulties by synthesizing l‐azidohomoalanine (Aha) – a ncAA containing an azido moiety – within *Escherichia coli* cells, thanks to the presence of an orthogonal recombinant PLP‐dependent enzyme from *Corynebacterium glutamicum*. In this system, Aha is inserted into the target proteins instead of methionine through the selective pressure incorporation method (SPI).^[7]^ This method was inspired from early work of Maier,^[21]^ in which an Aha analogue (L‐azidoalanine) was produced by a cultivation‐based approach after re‐engineering the cysteine biosynthetic pathway in *E. coli*. Generally speaking, Aha, like several other methionine analogues, is simply introduced into recombinant proteins taking advantage of Met‐auxotrophic *E. coli* strains;^[22]^ this can be done for a variety of biotechnological purposes. One of the best‐known examples is the use of seleno‐Met derivatives for phase resolution in protein crystallization, by means of multiwavelength anomalous diffraction techniques.^[23]^ In the last decade, Aha has been established as one of the most important bio‐orthogonal tags in chemical and synthetic biology. Bio‐orthogonal noncanonical amino acid tagging (BONCAT), is a very versatile methodology. It can be used on microbial and eukaryotic cultured cells, subpopulations of cells in complex multicellular eukaryotes, and even native plant tissues,^[24]^ and is particularly suitable when direct labeling, radioisotope labeling, or the use of antibodies is not applicable or inefficient. In this contest, Aha bio‐orthogonal tagging has been successfully used, for example, for the detection of newly synthesized proteins and quantification of protein degradation.[^25^, ^26^, ^27^, ^28^, ^29^, ^30^, ^31^] Other natural and unnatural Met analogues with different chemical characteristics – such as trifluoromethionine, norleucine, homoproproargylglycine – have also been produced and incorporated into proteins for analytical, structural and functional studies on polypeptides and biopolymers, providing interesting applications in biological and medicinal chemistry, materials, environmental, and agrochemicals science.[^29^, ^32^, ^33^] Moreover, great efforts have been made to expand the possibility of residue‐specific incorporation when the wild‐type translation apparatus does not support the incorporation of long‐ or bulk‐chain noncanonical Met analogues such as azidonorleucine or trifluoronoreleucine; in this case, alteration of the biosynthetic machinery was required by the development of novel aminoacyl‐tRNA synthetases (aaRSs).[^34^, ^35^] In a previously described system for direct production and incorporation of Aha,^[7]^ the synthesis of the ncAA still required the addition of an exogenous amino acid precursor (i. e., *O*‐acetyl‐L‐homoserine). In the present paper, the system has been optimized by developing a novel, metabolically engineered *E. coli* strain with the addition of another building actor – namely, homoserine acetyltransferase (HSAT) from *C. glutamicum* – allowing in‐cell bio‐production of Aha through a trans‐sulfuration pathway, directly from primary cell metabolites, followed by incorporation of the NCAA into recombinant target proteins.

We demonstrate the feasibility of this approach by describing an auxotrophy‐based, residue‐specific method to introduce Aha into diverse model proteins such as barstar (B*), green fluorescent protein (GFP), enhanced cyan fluorescent protein (ECFP), and *Geobacillus thermoleovorans* lipase (GTL) variants, carrying from 1 to 7 methionine residues.

## Results and Discussion

### Prerequisites for intracellular production of l‐azidohomoalanine in *E. coli* host cells

**Scheme 1:**
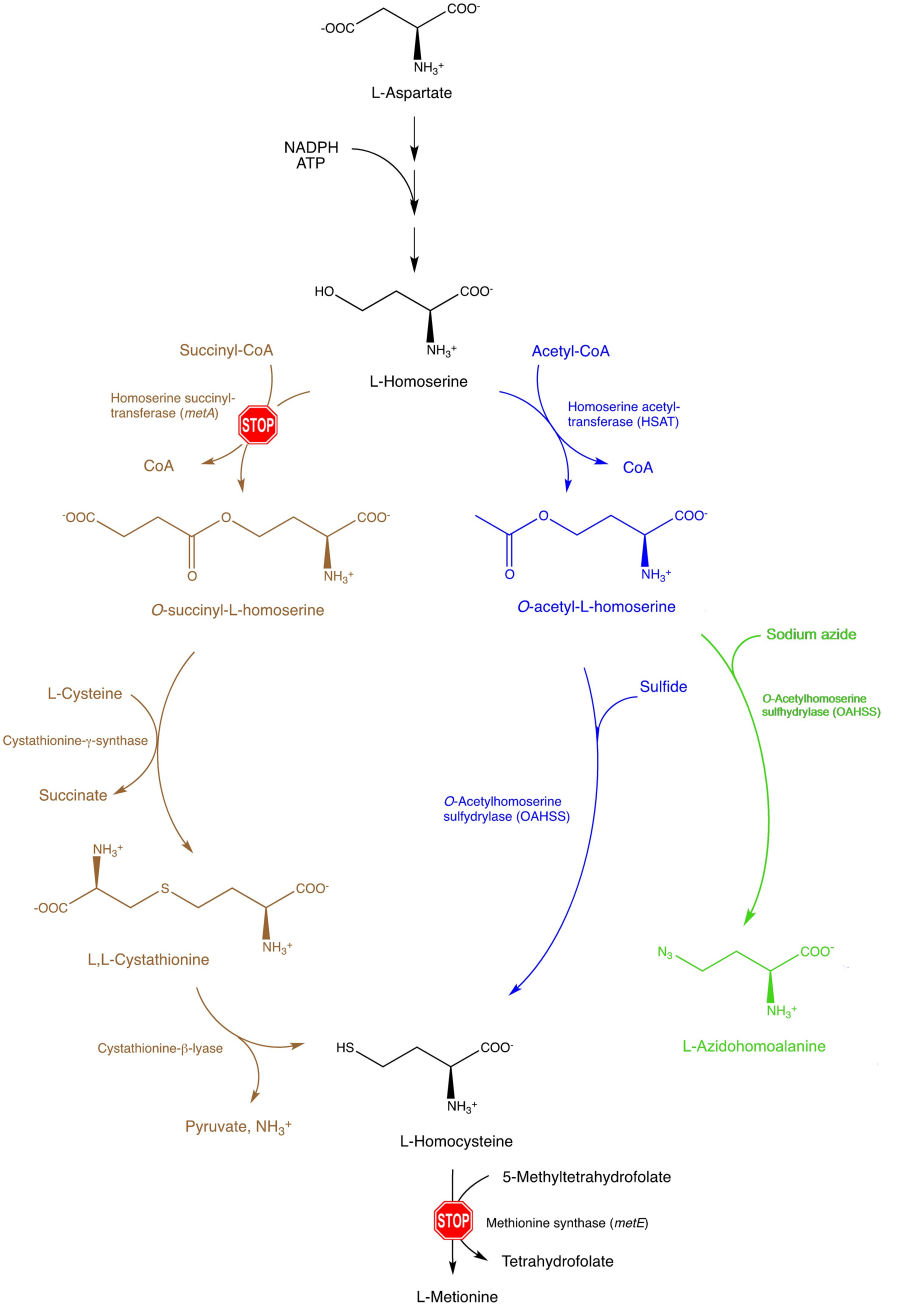
The engineering of a direct sulfhydrylation pathway in *E. coli* methionine metabolism. Scheme of the altered l‐methionine metabolism in engineered *MDS15* and *MDS15A E. coli* strains where *metA* and *metE* genes have been knocked out; direct sulfhydrylation pathway enzymes *cg*HSAT and *cg*OAHSS were introduced on a pSEVA26′*glnS* plasmid for production of Aha. The *E. coli* strain JW3973 (Met‐auxotrophy control) has only the *metA* gene knocked out. The trans‐sulfuration pathway is shown in brown, direct sulfhydrylation pathway in blue, L‐azidohomoalanine production in green.

L‐Azidohomoalanine (Aha) can be produced enzymatically, starting from l‐homoserine, by means of two subsequent reactions catalyzed by HSAT and *O*‐acetyl homoserine sulfhydrylase (OAHSS). These two enzymes belong to the so‐called direct sulfhydrylation pathway (Scheme 1, blue branch). This pathway is present in the Gram‐positive soil bacterium *C. glutamicum*, but is not present in *E. coli*, in which l‐homocysteine (hCys) is formed – as methionine precursor – through a different metabolic pathway, that is, trans‐sulfuration (Scheme 1, brown branch). It was previously shown that OAHSS displays a relaxed substrate specificity,[^36^, ^37^, ^38^] accepting several types of thiols and selenols as substrate nucleophiles, as well as azide, cyanide and aromatic five‐membered heterocycles containing at least two neighboring nitrogen atoms.^[21]^ In our enzymatic reaction scheme, OAHSS from *C. glutamicum* will react with the unnatural substrate sodium azide (NaN_3_), instead of sulfide, leading to Aha production in place of methionine (Scheme 1, green branch).

#### Metabolic engineering of *E. coli* strains for l‐azidohomoalanine production

In our system, the enzymatic reaction scheme described above was exploited in *E. coli* to generate a bacterial strain for the production of Aha directly from primary metabolites, that is, from normal growth medium without supplementation of exogenous expensive reagents. This is an important step forward with respect to our previously published system,^[7]^ which still required the addition of exogenous *O*‐acetyl‐L‐homoserine as precursor. In order to achieve our goal, a metabolically engineered *E. coli* strain was generated, with the following features:

1. methionine auxotrophy: two essential genes of the methionine biosynthetic pathway (*metA* and *metE)* were knocked out (STOP signs in Scheme 1); in this new strain, l‐homoserine produced from the aspartate pathway cannot be succinylated by homoserine succinyltransferase (*metA*), and consequently will not enter the trans‐sulfuration pathway. Furthermore, any l‐homocysteine produced through direct sulfhydrylation by OAHSS upon reaction with sulfide cannot be transformed into l‐methionine, because also methionine synthase (*metE*) has been knocked out;
2. presence of direct sulfhydrylation pathway enzymes (Scheme 1, blue branch): the coding regions of HSAT and OAHSS from *C. glutamicum* were introduced into the *E. coli* strain through a plasmid, under control of a strong *E. coli* promoter (*glnS*). When sodium azide is added to the culture medium, this metabolically engineered strain will produce l‐azidohomoalanine from l‐homoserine (Scheme 1, green branch).

**Figure 1:**
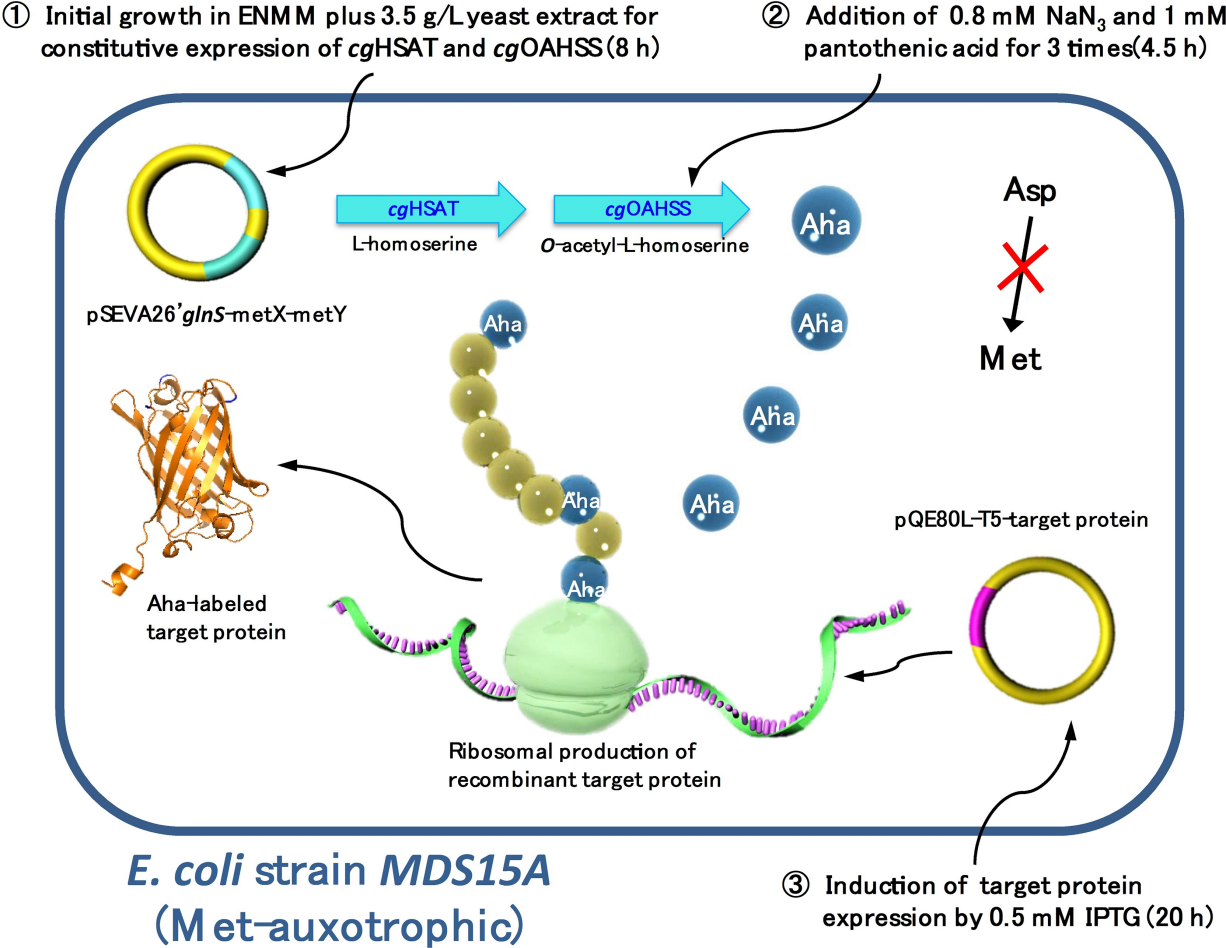
Intracellular metabolic pathway for direct Aha synthesis and incorporation. The engineered Met‐auxotrophic *E. coli MDS15* and *MDS15A* host strains are transformed with two different plasmids: one is pSEVA26′*glnS*‐*metY*‐*metX*, carrying the genes for *cg*HSAT and *cg*OAHSS expression, under the control of constitutive promoter *glnS*; the other one is pQE80L, carrying the cDNA target protein under inducible expression of the T5 promoter. The metabolic pathway for Aha intracellular biosynthesis and incorporation into target proteins can be described in three steps: 1) Initially, 3.5 g/L yeast extract is supplied to the recombinant auxotrophic strain as a methionine source to get the *metX* and *metY* genes constitutively expressed; at the end of this phase, methionine in the medium should be exhausted. 2) Sodium azide (0.8 mM) and pantothenic acid (1 mM) are fed to the culture three times to ensure Aha intercellular synthesis and accumulation. 3) The expression of target proteins is induced by 0.5 mM IPTG, and Aha is incorporated in place of Met by AUG codon reassignment.

This new *E. coli* strain was derived from commercially available *B834(DE3)* (a Δ*metE E. coli* strain) by knocking out the gene encoding for homoserine succinyltransferase (*metA*). The knockout resulted in a new strain, named *MDS15* (genotype: F^−^ *ompT hsdS_B_(r_B_* ^*−*^ *m_B_^−^) gal dcm met*(ΔE3) *ΔmetA)*. In this strain, *C. glutamicum* homoserine acetyltransferase (*cg*HSAT) and *C. glutamicum O*‐acetyl homoserine sulfhydrylase (*cg*OAHSS) are constitutively expressed through transformation with the plasmid construct pSEVA26′*glnS*‐*metY*‐*metX* (Table S1). The overall picture for the direct Aha production and incorporation into proteins is shown in Figure 1.

We also attempted genetic improvement of this new *MDS15* strain through directed evolution, in order to achieve a more robust growth that would possibly improve expression of model proteins. After about 27 days growth in a Genemate 3 turbidostat apparatus (see the Experimental Section for conditions), the starting *E. coli* strain MDS15 reduced its initial generation time by about 40 % and stayed at a plateau (Figure S1). This new adapted strain was named *MDS15A*. Both strains were checked for tolerance toward the toxic substrate sodium azide. *MDS15* and *MDS15A* were grown on new minimal medium (NMM; see the Experimental Section) with the addition of different concentrations of sodium azide, varying from 0.5 to 1.5 mM. Viable cell count was determined by serial dilution and incubation and is shown in Table 1. Under these experimental conditions, 0.8 mM sodium azide appears as a good compromise between a robust cell growth and a high azide concentration needed to sustain the production of Aha. Therefore, we decided to use a concentration of 0.8 mM sodium azide for our future experiments. Although the tolerance to sodium azide was not improved by directed evolution, the growth ability in the presence of 0.8 mM sodium azide has become better for the evolved strain *MDS15A* compared to *MDS15*. Noteworthy, the fact that at this concentration of sodium azide cells were able to survive and grow, demonstrates the principle of conversion of an otherwise toxic compound through the incorporation into a customized metabolic pathway.

**Table 1:** Cell counts of different dilutions of *MDS15* and *MDS15A*, grown in NMM medium for different lengths of time with increasing concentrations of sodium azide.

[Sodium azide]	Cell count [cells/mL]			
	MDS15		MDS15A	
	3.5 h	6 h	3.5 h	6 h
0.5 mM	7×10^7^	5×10^8^	9×10^7^	6×10^8^
0.8 mM	2×10^7^	1×10^7^	4×10^7^	3×10^7^
1.0 mM	2×10^6^	1×10^6^	3×10^5^	1×10^5^
1.5 mM	1×10^5^	1×10^5^	5×10^4^	3×10^4^

#### Met‐auxotrophy survival test to determine the activity of direct sulfhydrylation enzymes

**Figure 2:**
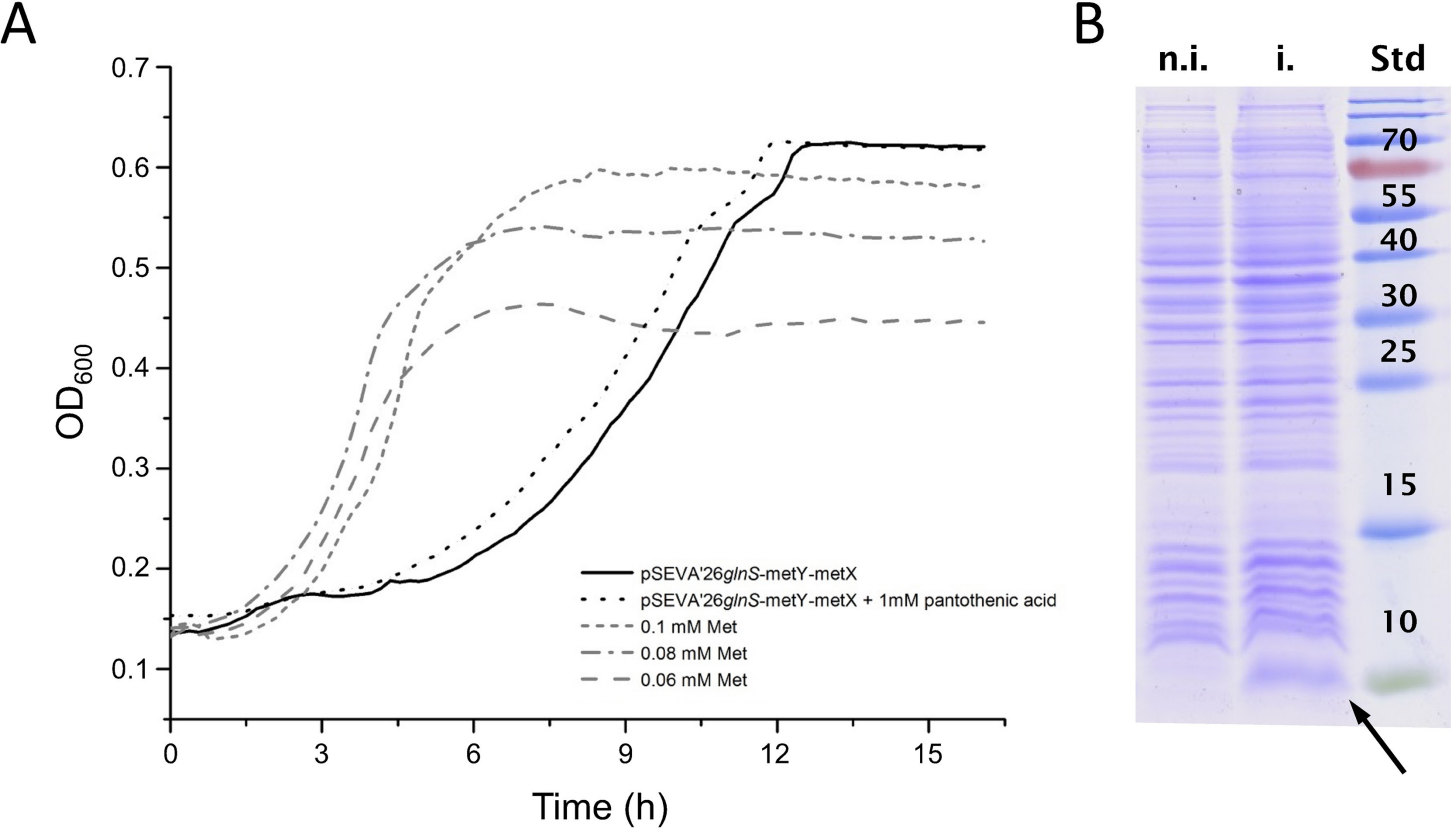
A) Growth curves for *E. coli* JW3973 with different setups and B) testing Barstar over‐expression as target protein. A) *E. coli* JW3937 with pSEVA′26*glnS*‐*metX‐metY* was grown in NMM without Met, and with (black dotted line) or without 1 mM pantothenic acid (black solid line). Control JW3973 was cultivated in NMM with 0.06, 0.08, and 0.1 mM Met (grey dotted lines, see legend in the figure). The cell culture level of *E. coli* JW3937 with pSEVA′26*glnS*‐*metX‐metY* reached a similar level to the control JW3973 cultured with 0.1 mM Met, although a longer lag phase was evident. The addition of 1 mM pantothenic acid to *E. coli* JW3937 with pSEVA′26*glnS*‐*metX‐metY* was able to slightly reduce this lag phase, but did not increase the final culture density; B) Non‐induced (n.i.) and induced (i.) cell extract from strain JW3973 with pSEVA26′*glnS*‐*metY‐metX* and pQE80L‐B*. A clear abundant band corresponding to B* (≈10 kDa, see black arrow. Std, molecular weight standards; molecular weight [kDa] is indicated in the picture) after expression induction with IPTG demonstrated that the biosynthesis of Met, based on *cg*HSAT and *cg*OAHSS in the Met‐auxotrophic strain JW3973, was enough to sustain over‐production of the induced target B*.

It was essential to know whether the enzymes introduced by the construct pSEVA26′*glnS*‐*metY*‐*metX* were catalytically active during the growth of the metabolically engineered *MDS15* and *MDS15A* strains. For this purpose, we designed a survival test system based on the methionine auxotrophic cognate strain *E. coli* JW3973, characterized by a deletion on the *metA* gene coding for homoserine *O*‐succinyltransferase essential for methionine biosynthesis (by comparison, *MDS15* and *MDS15A* carry deletions at both *metA* and *metE* genes). Due to its auxotrophy, JW3973 growth is limited by the Met concentration in the cultivation medium. On the other hand, when JW3973 strain is complemented with the addition of the trans‐sulfuration pathway enzymes (HSAT and OAHSS from *C. glutamicum*), Met biosynthesis can be recovered as hSer→Oahs→hCys→Met (hSer=l‐homoserine, Oahs=*O*‐acetyl‐L‐homoserine; Scheme 1); thus, the JW3973 strain harboring pSEVA26′*glnS*‐*metY‐metX* should be able to survive in Met‐lacking NMM medium using its self‐synthesized Met through the constitutively expressed *cg*HSAT and *cg*OAHSS. Only successfully expressed *cg*HSAT and *cg*OAHSS – with substantial catalytic activity – will allow JW3973 to survive in media without Met. The optical density reached by JW3973/pSEVA26′*glnS*‐*metY*‐*metX* can be used to indirectly estimate the amount of Met synthesized (and thus, also its downstream precursor homocysteine) by comparing it to a control experiment in which JW3973 is grown in NMM with addition of Met. Moreover, to improve the intracellular Oahs production during cell growth, we tested the effect of pantothenic acid, a precursor of acetyl‐CoA, which is essential for the formation of Oahs from hSer catalyzed by *cg*HSAT. Therefore, the JW3973 survival test system may prove to be an efficient approach not only for the measuring the catalytic functionality of *cg*HSAT and *cg*OAHSS in the *E. coli* host, but also to estimating intracellular hSer levels. As shown in Figure 2A, the growth of JW3973 with *cg*HSAT and *cg*OAHSS reached about the same level as the control JW3973 with up to 0.1 mM Met. This means that a comparable concentration of hSer precursor was able to contribute to the Met biosynthesis – and can be used for Aha synthesis in our *MDS15* and *MDS15A* double mutant strains. Additionally, pantothenic acid was able to slightly improve the specific growth rate of the cells. Besides demonstrating that the activity of *cg*HSAT and *cg*OAHSS was able to rescue JW3973 cell growth, the survival test described above was also used to verify that the recovered Met biosynthesis pathway was able to produce enough methionine to be incorporated into over‐expressed target proteins, as shown in Figure 2B for the over‐expression of B* from the IPTG inducible plasmid pQE80L.

#### Optimization of growth and protein expression conditions

**Figure 3:**
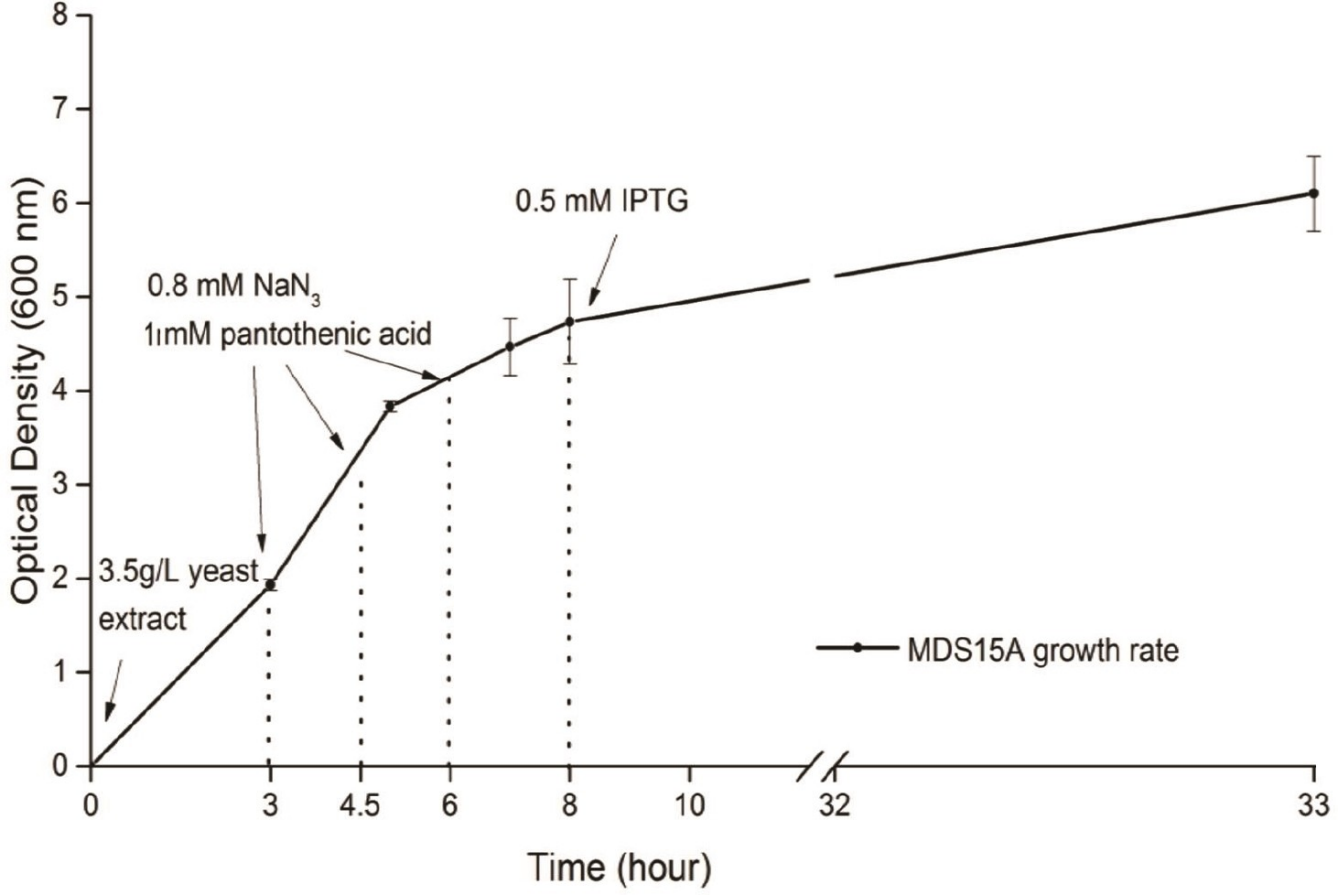
Experimental protocol for the growth and expression of Aha‐labeled target proteins in *MDS15* and *MDS15A* host strains. The addition of 3.5 g/L yeast extract to the medium leads to OD_600_≈4.5 after about 8 h at 37 °C. After 3 h, 0.8 mM sodium azide and 0.8 mM pantothenic acid were fed to the cultures every 75 min, for a total of three times. After 8 h, 0.5 mM IPTG was added for induction of target protein expression, and the growth was allowed to continue at 28 °C for about 25 h. In the figure, the experimental growth rate diagram of *MDS15A* is shown.

Normally, when using the SPI method for the production of ncAA‐substituted proteins, the required amount of noncanonical amino acid added to the culture medium varies from 0.5 to 1 mM. Although not all of the supplemented ncAA will actually be taken up and used by the cell, it is still necessary to guarantee a high intracellular ncAA concentration compared to canonical amino acids, so that the protein synthesis machinery prefers to incorporate the ncAA.^[39]^ Thus, it is essential to optimize the cultivation protocol to efficiently produce enough desired ncAA inside the cell. Within the cultivation scheme shown in Figure 1, the expression of the desired ncAA‐substituted target protein can be evaluated with two different criteria: protein yield and methionine replacement yield. The protein yield is mainly driven by cell mass, media conditions, and efficiency of translation, whereas the labeling yield relies on the accumulated noncanonical amino acid concentration. In contrast from normal SPI experimental set‐ups, where the ncAA is added to the culture in high concentration at once, our system based on *MDS15* and *MDS15A* expression hosts requires a longer time to accumulate the ncAA inside the cell before the production of target protein should be induced. A diagram of our growth/expression system is shown in Figure 3. In the first phase of the bacterial growth, a limited amount of Met has to be added to the culture medium in order to reach a high cell density. As a cheap and rich nutrition source, yeast extract can be added instead of purified Met for this first phase^[40]^ and thus a cell density of OD_600_≈4.5 can be reached within a cultivation time of 8 hours. This high cell density requires a good pH buffering capacity of the culture medium; for this reason, the concentration of the phosphate buffer was increased by 2.5 times in the enhanced new minimal medium (ENMM) compared to the original NMM (see the Experimental Section). Three hours after the start of the cultivation, 0.8 mM sodium azide and 0.8 mM pantothenic acid were added to the growing medium every 75 minutes, for a total of three additions. After 8 hours, the induction of the target protein expression was initiated by the addition of 0.5 mM IPTG and the cultivation was continued for 25 hours. Unlike the canonical SPI methods, the induction phase must be under strict control due to the intrinsically low ncAA concentration and the long cultivation time. This could be reached by a combination of lower IPTG concentration, shorter induction time, and the use of media which provide a slow release of glucose.^[41]^ On the other way, increasing IPTG concentration to 1 mM will lead to higher protein expression, but lower labeling yield (data not shown).

### Incorporation of l‐azidohomoalanine produced in‐cell into a selection of over‐expressed target proteins

Having obtained a robust and optimized cellular system for self‐sustainable intracellular Aha synthesis in minimal medium, the next step was to demonstrate its performance in the expression of various recombinant target proteins. Several target proteins were chosen for proving the feasibility of the system. Proteins were chosen on the basis of their solubility, ease of purification, suitability in terms of structure and spectroscopic analysis, biotechnological interest and number of methionine residues present in the primary structure. Of particular importance was to test the performance of the system towards multiple replacements of Met residues. To facilitate purification, all target proteins (except barstar) were added with a His_6_ tag which, when positioned at the N‐terminal end, can be subsequently cleaved by tobacco etch virus nuclear‐inclusion endopeptidase (TEV protease), giving rise to a protein sequence without the original N‐terminal Met residue. The proteins object of our studies were:

Barstar (B*): a small protein composed of 90 amino acids found in *Bacillus amyloliquefaciens* as the inhibitor of barnase. Barstar was used as test protein in our previous system for production and incorporation of Aha.^[7]^ The protein used in this paper contains two Met residues, at positions 1 and 47;

*Green fluorescent protein*: a protein from the jellyfish *Aequorea victoria* widely applied in biotechnology. We used a modified form of the sfGFP version engineered for protein robustness.^[42]^ Our adapted sfGFP version contains an N‐terminal His_6_ tag and a TEV cleavage site, and is present in two variants: GFP1M (with a D134M mutation, containing only one Met residue, at position 134); GFP2M with one additional Met residue, that is, mutation T50M together with D134M. In both variants, the N‐terminal Met is removed simultaneously with the His_6_ tag by post‐purification TEV digestion

*Enhanced cyan fluorescent protein*: a color mutation of GFP with chromophore Tyr66 replaced with tryptophan; it is also a powerful tool for protein analysis. We used two mutants employed in earlier studies.^[43]^ The first one is ECFP‐C, which contains five Met residues and a C‐terminal His_6_ tag. In this variant, due to the presence of a valine residue in second position in the sequence, the N‐terminal Met is partially cleaved from the protein structure by intracellular Met aminopeptidases;[^44^, ^45^] the other ECFP variant is ECFP‐N, which contains a N‐terminal His_6_ tag (lacking TEV cleavage site) and Met residues in six different positions (namely, 1, 13, 91, 101, 231, 246). In this case, a bulky Arg residue is present in second position in the sequence, preventing the cleavage of the N‐terminal Met.^[46]^

Geobacillus thermocatenulatus *lipase* (*GTL*): thermostable lipases are commercially important industrial enzyme catalysts; this protein contains seven methionine residues in its primary structure, but after TEV cleavage only six Met residues remain.

The complete sequences of all above‐mentioned proteins are shown in Figure S2.

During the whole optimization process, a variety of parameters were taken into consideration. A scheme of the different experimental setups is shown in Table 2. The investigated parameters included host strain, Met source, length of its initial consumption phase, sodium azide and pantothenic acid feeding strategy, target protein type and induction time. The success of the whole cultivation process was determined by protein yield and Aha labeling yield.

**Table 2:** Chart of fermentation conditions and protein yields. Host refers to the host *E. coli* strain for fermentation.

**Setup**	**Model protein**	**Host^[a]^**	**Met source^[b]^**	**Azide^[c]^**	**PA^[d]^**	**Ind. time^[e]^**	**Protein yield^[f]^ [mg/L]**	**Label yield^[g]^**
1	B*	*M*	0.06 mM Met, 8 h	0.8 mM, 1 h	–	o/n	1.5	1/2
**2**	**GTL**	***M***	**0.06 mM Met, 8 h**	**0.8 mM, 1 h**	**–**	**o/n**	**5.0**	**6/7**
3	GTL	*M*	0.06 mM Met, o/n	0.8 mM, 3 h	–	5 h	1.5	5/7
4	ECFP‐N	*A*	0.06 mM Met, 7 h	0.8 mM, 1 h	–	o/n	14.1	3/6
5	ECFP‐C	*A*	0.06 mM Met, 6 h	0.8 mM, 3 h	–	o/n	2.6	3/5
6	GFP‐1M	*A*	0.06 mM Met, 7 h	0.8 mM, 1 h	–	o/n	2.7	0/1
7	GFP‐2M	*A*	0.06 mM Met, 7 h	0.8 mM, 1 h	–	o/n	5.7	1/2
**8**	**ECFP‐N**	***A***	**3.5 g/L YE, 8 h**	**2.4 mM, 4.5 h** ^[h]^	**1** mM	**20 h**	**36.0**	**5/6**
**9**	**GFP‐1M**	***A***	**3.5 g/L YE, 8 h**	**2.4 mM, 4.5 h** ^[h]^	**1** mM	**20 h**	**9.7**	**1/1**
**10**	**GFP‐2M**	***A***	**3.5 g/L YE, 8 h**	**2.4 mM, 4.5 h** ^[h]^	**1** mM	**20 h**	**20.0**	**2/2**
11	ECFP‐N	*A*	3.5 g/L YE, 8 h	5.6 mM, 4.5 h^[i]^	–	20 h	14.0	1/6

[a] *M*: *MDS15* and *A*: *MDS15A*; [b] The methionine source and its feeding time; [c] The feeding strategy of sodium azide; [d] Addition of pantothenic acid. [e] Length of induction time. [f] Amount of purified protein, expressed in mg per L culture. [g] Detected Aha residues divided by total Met positions in the target proteins, as determined by MS analysis (In the table, yield results should be taken as a rough estimate; a more thoroughly analysis of the labeling yield was performed by dOG conjugation and is discussed in the text). Protein and label yield values are the average of at least three determinations. The range of values was always within ±5 % from the average value. [h] For setups 8–10, supplying of 0.8 mM sodium azide was performed 3 times every 1.5 h. [i] For setup 11, 0.8 mM sodium azide was added 5 times. in **bold**, the most successful setups (described in the text).

**Figure 4:**
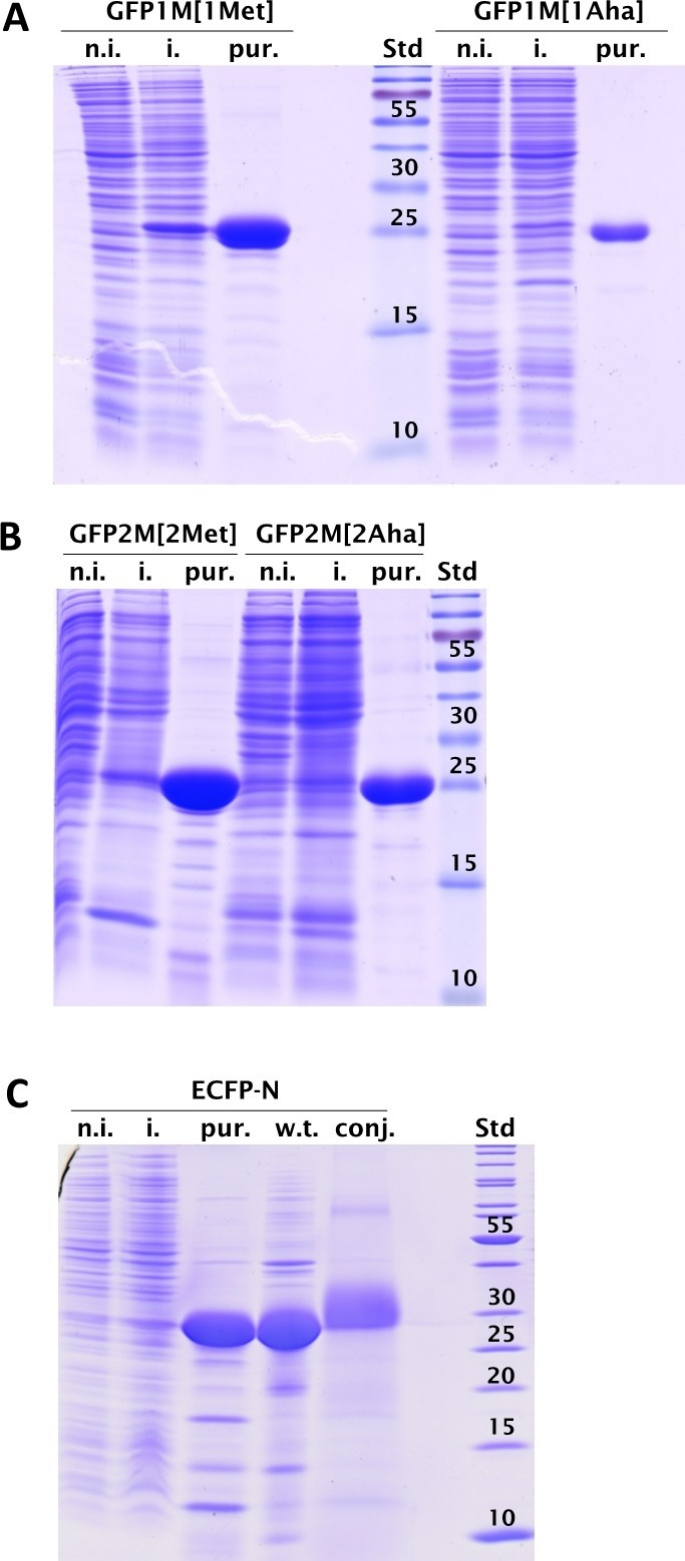
Coomassie‐stained 15 % SDS‐PAGE of GFP (A, B) and ECFP‐N (C) expression and purification. A) GFP1M grown in the presence of nonlimiting amounts of methionine, giving rise to GFP1M[1Met] and GFP1M grown according to our Aha production/incorporation protocol. B) GFP2M grown in the presence of nonlimiting amount of methionine, giving rise to GFP2M[2Met] and GFP2M grown according to our Aha production/incorporation protocol. The nominal molecular weight of GFP is 26.7 kDa. C) ECFP‐N grown according to our Aha production/incorporation protocol. The nominal molecular weight of ECFP is about 28.3 kDa. n.i., noninduced cell extract; i., induced cell extract; pur., purified protein; w.t., ECFP‐N grown in the presence of a nonlimiting amount of methionine; conj., dOG conjugated ECFP‐N, whose molecular weight is expected to be about 3 kDa higher than that of the untreated sample; Std, PageRuler marker with labeled molecular weights [kDa].

**Figure 5:**
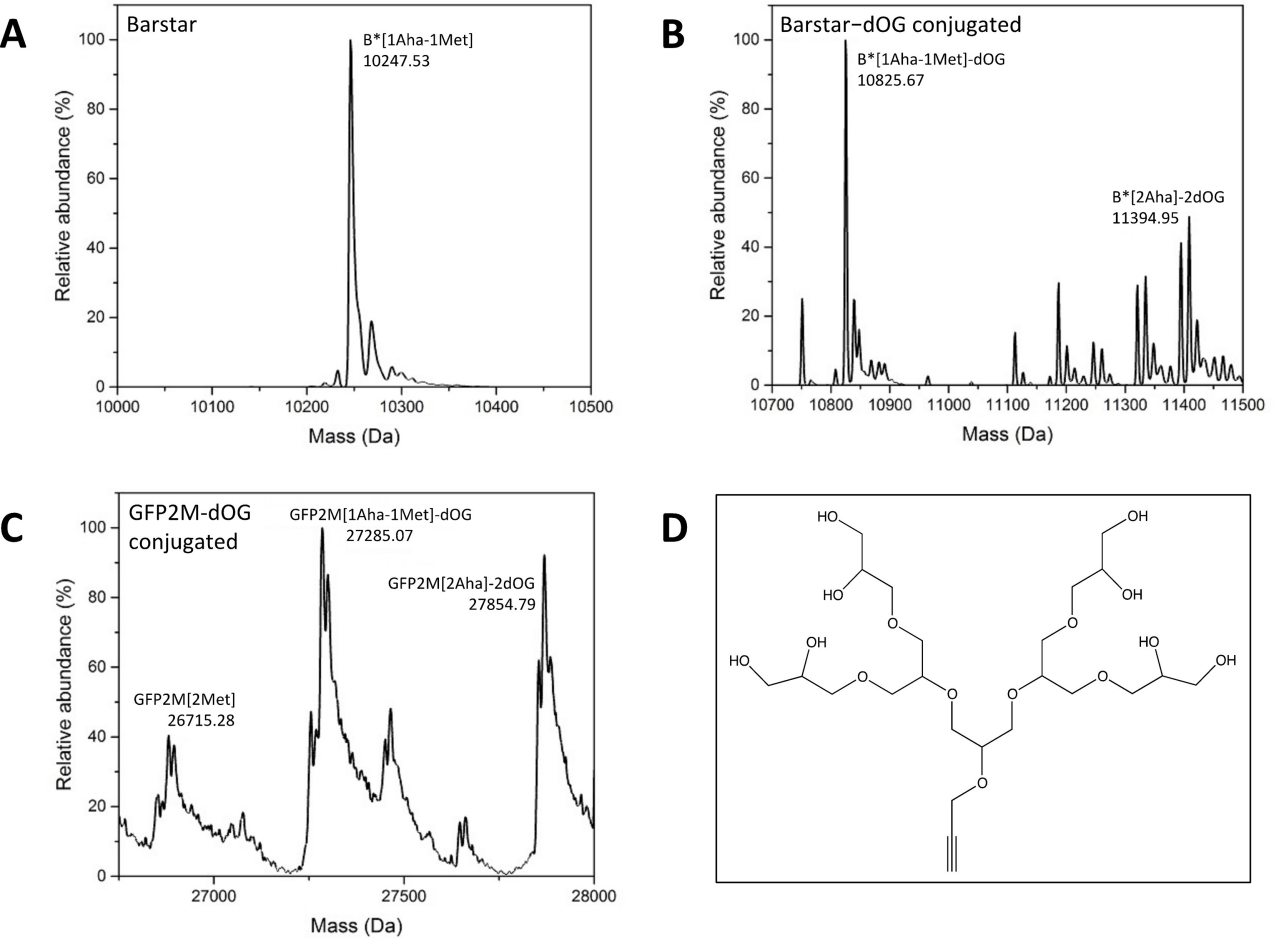
Mass spectrometry analysis of different target proteins and their dOG‐conjugated variants. A) B* expressed in *MDS15A*, according to our Aha production/incorporation system (setup number 1 in Table 2). B) same sample as in (A), after dOG conjugation. C) GFP2M expressed in *MDS15A* according to our Aha production/incorporation system (right; setup number 10 in Table 2), after dOG conjugation. The sample before dOG conjugation is shown in Figure S3B, right. D) Chemical structure of oligoglycerol dendrimer used in this work (molecular weight 574.28 Da). The values of deconvoluted and theoretical calculated masses are shown in Table 3. Mass spectrometry data of dOG‐conjugated Aha‐labeled proteins show wider and less distinct peaks, often accompanied with additional, peaks at regularly intervals – although a clear maximum density peak corresponding to the expected mass value can be recognized. This phenomenon can be attributed either to minor protein degradation during click reaction, or to dendrimer branch cleavage at ether bonds, consequent to acetic acid treatments of the samples prior to mass spectrometric analysis.

In Figure 4 are shown the SDS‐PAGEs of expression and purification steps of the experiments described in setups 8, 9 and 10 of Table 2, which represent the most successful of all trials. In all cases, a clear band corresponding to the expected molecular weight indicates high yield of target protein production. The results are compared to a control experiment in which 0.3 mM Met only were added to the culture medium (i. e., no sodium azide was added). Purified proteins were then analyzed by mass spectrometry in order to evaluate the Aha incorporation yield. In Figure S3, the mass spectrometry results for GFP1M and GFP2M are reported (setups number 9 and 10 in Table 2; Figure 5A and B, respectively. See also Table 3 for calculated mass values). The good correlation between the calculated and experimentally determined values of mass spectrometric analysis for GFP1M and GFP2M shows the successful incorporation of *in situ* synthesized Aha into both GFP variants. Figure S4 depicts the results for ECFP (ECFP‐C in Figure S4A and ECFP‐N in Figure S4B; setups number 5 and 8 in Table 2, respectively. See also Table 3 for calculated mass values). In the case of ECFP‐C, the setup was clearly not optimal for high Aha labeling (also the protein yield was quite low; data not shown). As it can be seen from the mass spectra, the peaks indicated a heterogeneous incorporation of Aha into the expressed protein, in which at most three Aha residues (out of a total of five) were incorporated. This protein also showed variations at the N‐terminal end, that was most likely occupied by a Met which was partially cleaved. The result was much better for ECFP‐N (setup 8) in which the single peak corresponding to 28260.07 Da shows that 5 Met residues over a total of six were substituted by Aha.

**Table 3:** Calculated values of deconvoluted and theoretical masses relative to the experiments shown in Figures S3, S4, and 5.

	Deconvoluted masses (Da)	Theoretical masses (Da) from the primary structure
Figure S3. Mass spectrometry analysis of different variants of GFP		
	GFP1M[1Met]: 26686.12 Da GFP1M[1Aha]: 26682.16 Da GFP2M[2Met]: 26716.26 Da GFP2M[2Aha]: 26707.57 Da	GFP1M[1Met]: 26685.80 Da GFP1M[1Aha]: 26680.72 Da GFP2M[2Met]: 26715.88 Da GFP2M[2Aha]: 26705.73 Da

Figure S4. Mass spectrometry analysis of different variants of ECFP		
ECFP‐C	ECFP‐C[5Met]: 27709.85 ECFP‐C[4Met]: 27577.97 ECFP‐C[3Aha‐2Met]: 27692.67 ECFP‐C[3Aha‐1Met]: 27561.77	ECFP‐C[5Met]: 27708.87 ECFP‐C[4Met]: 27577.68 ECFP‐C[3Aha‐2Met]: 27693.57 ECFP‐C[3Aha‐1Met]: 27562.38
ECFP‐N	ECFP‐N[6Met]: 28285.05 ECFP‐N[5Aha‐1Met]: 28260.07	ECFP‐N[6Met]: 28284.51 ECFP‐N[5Aha‐1Met]: 28259.01

Figure 5. Mass spectrometry analysis of different target proteins and their dOG‐conjugated variants		
B*	B* [1Aha‐1Met]: 10247.53 B* [1Aha‐1Met]‐dOG: 10825.67 Da B* [2Aha]‐2dOG: 11394.95	B* [1Aha‐1Met]: 10249.91 B* [2Met]: 10254.98 B* [2Aha]: 10244.83 B* [1Aha‐1Met]‐dOG: 10824.19 B* [2Aha]‐2dOG: 11393.39
**GFP**	GFP2M [2Met]: 26715.28 GFP2M [1Aha‐1Met]‐dOG: 27285.07 GFP2M [2Aha]‐2dOG: 27854.79	GFP2M [2Met]: 26715.88 GFP2M [1Aha‐1Met]‐dOG: 27285.09 GFP2M [2Aha]‐2dOG: 27854.29

Based on the results summarized in Table 2, the addition of 3.5 g/L yeast extract in the initial growth phase enabled the highest protein yield for each variant. Sodium azide and pantothenic acid continuous feeding (setups 8–10) brought the highest labeling levels. Also the effect of an increased concentration of sodium azide was studied (see setup 11). Although the cells were viable and it was still possible to harvest the target protein with good yields, the labeling level dropped dramatically (to 1 of a total of 6 positions). This suggests that too much sodium azide could interfere with the production and/or incorporation of Aha.

Attempts to express GTL were only performed by initial Met feeding, and also gave good results, especially considering the high number of Met residues present in this protein to be replaced by Aha. In the best of these setups (number 2 in Table 2), we were able to incorporate six Aha residues on a total of 7 Met present in the structure, although with a low yield of total protein (Figure S5).

Considering the variability of the outcomes (Table 2) – which might likewise be ascribed to the overall structure of the target protein – it is of paramount importance to find the right cultivation and expression parameters to assure a full consumption of the initially added Met added during the first phase (before induction), in order to obtain a high Aha production, and to achieve the best protein labeling yield. Nevertheless, absolute depletion of Met in the cultivation medium cannot be achieved, as found even with SPI methods.^[47]^ Together with the inevitable leakage of the T5 promoter in the first growth phase, this is responsible for small residual amounts of wild‐type or poorly labeled target protein. Beside the inaccuracy of the instruments, these variations could also be the reason of the slight discrepancy between the deconvoluted and expected values, which is in the range of 0.1–0.6 per ten thousand (‱) of the molecular weight. The molecular weight difference between Aha and Met is small, only 5.07 Da, and this may lead, during the deconvolution procedure of the mass spectrometric measurements, to an average value between the Aha‐ and the Met‐labeled target protein, if both are present in the same sample. For instance, in Figure S3A, the deconvoluted mass of GFP1M[1Aha], 26682.16 Da, shows a slightly higher molecular weight compared to the calculated GFP1M[1Aha] value, probably due to a minor contamination by GFP1M[1Met] in the protein mixture, which could be caused by both T5 promoter leakage and low concentration of *in situ* synthesized Aha.

#### Post‐synthetic modifications: Bio‐orthogonal oligoglycerol dendrimer conjugation with Aha‐labeled target proteins

Oligoglycerol dendrimer (dOG) is a very attractive type of branched glycerolpolymer to be used in bioconjugation field.^[48]^ To prove the functionality of incorporated Aha residues, the dOG moiety with an alkyne‐group (Figure 5D) was conjugated to different Aha‐labeled protein variants via copper catalyzed azide‐alkyne cycloaddition (CuAAC).^[49]^ Every dOG addition to each Aha residue results in a molecular weight increase of 574.28 Da, which can be easily verified by both SDS‐PAGE and mass spectrometry measurements. Especially in case of inhomogeneous target proteins, this significant mass shift caused by dOG conjugation can help estimate the incorporation yield of Aha‐labeled protein. In a first experiment, the ECFP‐N[5Aha‐1Met] variant (setup 8 in Table 2) was chosen for CuAAC with dOG. As shown in the SDS‐PAGE (Figure 4C, lane marked as conj.), a clear mass shift of approximately 3 kDa occurred upon dOG conjugation, compared with the purified ECFP‐N[5Aha‐1Met] (lane marked as pur.)

Further dOG conjugation experiments were then conducted with B* and GFP2M Aha‐labeled samples (setups 1 and 10 of Table 2, respectively; Figure 5A–C. See also Table S2 for calculated mass values). In Figure 5A, the deconvoluted mass of Aha‐labeled B* protein (before dOG conjugation) is shown to be 10247.53 kDa, which suggests the presence of either a mixture of B*[2Aha] and B*[2Met], or the presence of a singly labeled protein B*[1Aha‐1Met]. In this particular case, due to the small difference in molecular weigh between the different isoforms, the composition of the mixture cannot be accurately determined by means of mass spectroscopy. On the other hand, if the sample was conjugated with dOG (Figure 5B), it is clear that the most abundant protein form is the one containing only one Aha residue, with a small fraction of B*[2Aha] also present. This clarifies the unexpected mass of the inhomogeneous B* product from Figure 5A, in which the presence of a minor amount of B*[2Aha] was not detectable. In Figure 5C, the results of dOG conjugation with GFP2M (setup 10 in Table 2) are shown. Mass spectra before conjugation are depicted in Figure S3B and displayed an apparently homogeneous GFP2M[2Aha] protein form, although the measured molecular weight is slightly higher than the theoretical one. In fact, after dOG conjugation three different constituents can be distinguish: beside a small fraction of protein sample containing two Met residues (GFP2M[2Met]), a mixture of GFP2M[1Aha‐1Met]‐dOG and GFP2M[2Aha]‐2dOG was present. These results suggest that, even in this case, the target protein was expressed in an inhomogeneous manner, resulting in three different variants that were not easily detectable before the conjugation experiments.

### Implementation and versatility of the system

Although full homogeneity of Aha‐labeled target proteins was not reached in all samples, our system can be considered as proof of concept for direct in‐cell production and incorporation of noncanonical amino acids. For many biotechnological applications, absolute homogeneity of the samples might not be required and one can envisage a subsequent purification step after sample conjugation.

An important feature of our methodology is the versatility of the system, which can be employed to produce and incorporate a number of homoserine‐based ncAAs, resulting from the particular nucleophile added to the culture medium. The key point of this feature is the ability of *C. glutamicum* OAHSS to accept different substrates, a characteristic known as “enzymatic promiscuity” a typical feature of PLP‐dependent enzymes.[^21^, ^50^, ^51^, ^52^] As a matter of fact, other PLP‐dependent enzymes able to perform the biosynthesis of amino acid analogues could be exploited in a similar way, keeping in mind that these hypothetical ncAAs have to be accepted as substrates at aminoacyl‐tRNA synthetase level and efficiently incorporated. Furthermore, it would be remarkable to further improve our system as to specifically incorporate ncAAs at defined positions via stop codon reassignment.^[19]^ A list of potentially interesting nucleophiles were tested *in vitro* as substrate for *cg*OAHSS and a number of them produced stable l‐homoserine derivatives (Table S4). For example, incorporation into proteins of ncAAs carrying a thioallyl group, such as *S*‐allyl‐L‐homocysteine can be of great interest.^[53]^ At a biotechnology application level, the benefit of alkene moieties present in thioallyl residues introduced into recombinant protein concerns the photochemical surface modification by both thiol‐ene click reaction[^54^, ^55^] and photoirradiation.^[56]^ These photochemistry click reactions are highly attractive bio‐conjugation methods because of the low harm for proteins in living systems. In addition, the thioallyl group offers the possibility for protein bioconjugation cross‐metathesis, carried out via catalysis of Hoveyda‐Grubbs under aqueous conditions.[^57^, ^58^]

## Conclusions

In modern biotechnology, great effort is directed towards engineering bioavailable and highly specific biomaterials, peptides and proteins with desirable properties for commercialization as pharmaceuticals and for diagnostic purposes. Recent research advances have provided solid evidence that the natural protein translation machinery can be reprogrammed to genetically encode a vast number of noncanonical amino acids. Chemical diversity gained in this way will allow for a dramatic increase in the scope of protein biosynthesis, and enable the expression of peptide‐based biomaterials having properties not found in nature. Recently, though, it has been argued that the limited access to reprogrammed protein translation to metabolically produced ncAAs is an Achilles′ heel for the whole field, and hinders its broader application in biotechnology.^[59]^ Current technologies for the expression of custom‐made proteins and peptides require ready‐synthesized unnatural amino acids. In most cases, their chemical or chemo‐enzymatic syntheses are complicated and expensive.

In this paper, we have described an innovative bacterial‐based methodology for the in‐cell synthesis and direct incorporation of l‐azidohomoalanine – in place of l‐methionine – into recombinantly expressed target proteins. The system takes advantage of a newly engineered bacterial strain, which allows for l‐azidohomoalanine production from primary metabolites (i. e., TCA cycle and amino acid intermediates) without need of addition of any metabolic precursor to the cell. The production rate of the ncAA is able to sustain cell growth and protein production to a level comparable to cells cultivated in the presence of nonlimiting amounts of l‐methionine.

The fundamental novelty of our technology opens the possibility to develop an integrated cell system capable of metabolically generating different kind of unnatural amino acids, and subsequently incorporating them into peptides, peptidomimetics, biomaterials and recombinant proteins within the same host cells. This will essentially require water, salts, trace elements and simple carbon sources. Our project is inspired by the concept of green, sustainable chemistry, trying to minimize the use and generation of hazardous substances, pushing towards enzyme chemistry and biotechnology‐based production. Note, the fact that basically toxic compounds – such as azides – can be used by the cell as a source of bio‐orthogonal amino acids is a clear example of an important evolutionary principle, that is, how living cells not only detoxify harmful compounds, but are actually able to convert them into useful metabolites.

Our system might indeed prove to be versatile in terms of the selected ncAA to be synthesized by the cell, and we expected it to be up‐scalable for production in large‐scale production bioreactors. Possible future applications may range from protein engineering, protein labeling, enzyme immobilization, peptide derivatization, peptidomimetics and peptidic antibiotic production for biotechnology, pharmaceutical and innovative material industry. Therefore, expanding the scope of protein biosynthesis with novel metabolically generated building blocks is highly relevant for future technologies, with classical chemical synthesis methods largely replaced by biotechnological processes.

## Experimental Section

**Chemicals**: All chemicals were purchased from Sigma‐Aldrich (Taufkirchen), Carl Roth GmbH (Karlsruhe), Merck (Darmstadt), or VWR International GmbH (Darmstadt).

**Molecular biology reagents**: GeneJETTM Plasmid Mini‐prep Kit, GeneJETTM PCR purification Kit, and GeneJETTM Gel extraction Kit were from Thermo Scientific. Phusion High‐Fidelity DNA Polymerase, dNTP mix, FastDigest restriction enzymes, and T4 ligase were from Thermo Scientific. Chicken egg white lysozyme and bovine pancreas ribonuclease (RNase A) were from Carl Roth (Karlsruhe). Bovine pancreas deoxyribonucleic (DNase I) was from Sigma‐Aldrich. TEV protease was self‐prepared.^[60]^ HiTrap Q‐sepharose 1 mL column and HisTrap FF Crude 1 mL column were from GE healthcare. Oligonucleotides were purchased from Biomers (Ulm) or Sigma‐Aldrich as desalted form. Primers longer than 40 bp were purchased in HPLC‐purity form. Acetyl‐CoA sodium salt was purchased from Sigma‐Aldrich. Silica Gel 60 F254 aluminum sheets were from Merck.

**Media and supplements**: New minimal medium (NMM)^[61]^ is composed by basic salts, plus additional nutrition elements. Basic salts contain: 7.5 mM (NH_4_)_2_SO_4_, 50 mM K_2_HPO_4_ and 22 mM KH_2_PO_4_, 8.5 mM NaCl, 1 mM MgSO_4_, pH 7.2; additional nutrition elements contain 20 mM d‐glucose, 50 mg/L of each canonical amino acid except Met, 1 μg/mL FeCl_2_, 1 μg/mL CaCl_2_, 10 μg/mL thiamine, 10 μg/mL biotin, 0.01 μg/mL trace elements (CuSO_4_, ZnCl_2_, MnCl_2_, (NH_4_)_2_MoO_4_). The enhanced new minimal medium (ENMM) is also used in this work, with similar components to NMM except for the higher concentration of buffering system (125 mM K_2_HPO_4_, 55 mM KH_2_PO_4_). Other compound for growing media were used at the following final concentrations: 0.045 mM l‐methionine, 1 mM pantothenic acid, 0.8 mM sodium azide, 0.5 mM isopropyl β‐d‐1‐thiogalactopyranoside (IPTG), 100 μg/mL ampicillin, 50 μg/mL kanamycin, 3.5 g/L yeast extract (note: quality of yeast extract may vary greatly; in this work, powered yeast extract for bacteriology, Carl Roth art. no. 2363.1, was used).

**Bacterial strains**: Bacterial strains used in this work are described in Table S1. *MDS15* and *MDS15A* were generated by *metA* gene knock‐out starting from *E. coli B834*(DE3). *MetA* was replaced by a FRT‐KanR‐FRT cassettes using the procedure described by Datsenko and Wanner^[62]^ and the primers metA‐P1 and metA‐P2 (Table S2). The evolution of MDS15 strain was performed in a Genemate 3 directed evolution automat (GM3)^[63]^ from Heurisko GmbH (Leipzig) commercialized by Altar (www.altar.bio) in continuous cultivation for 27 days. The cultivation conditions for turbidostat mode in NMM with additional 0.06 mM l‐methionine were set at 30 °C, twice a day sterilization with 6 M NaOH and pulses with 30 % of the culture volume by fresh NMM to keep the biomass to a fixed point (OD at 600 nm around 0.8). The generation time (*t*) was calculated based on the pulses number (*n*) per day with the following formula:

$$t=\frac{\ln\;2\times 60\times 24\times 3}{n}$$

**Plasmid construction**: Construction of pSEVA26′*glnS*‐*metY‐metX*. The sequence of constitutive *glnS′* promoter was amplified by PCR (see primer list in Table S2 for oligonucleotide sequences) together with *C. glutamicum metY* gene which coded for *cg*OAHSS from previously constructed plasmid pBU26′1GK‐*metY*‐HTC^[7]^ and inserted into the plasmid pSEVA26′1 between AvrII and SmaI restriction sites.^[64]^ From the template plasmid pZ8‐1metX (provided by Prof. Jörn Kalinowski's lab, Bielefeld University, Germany), the *metX* gene sequence encoding *cg*HAST was amplified and inserted into the same plasmid between SmaI and XbaI restriction sites.

Besides the pSEVA26′*glnS*‐*metY‐metX* construct for expression of *cg*HSAT and *cg*OAHSS, our system also involved vectors for target protein overexpression. cDNAs encoding for target proteins were PCR‐amplified and cloned into the expression plasmid pQE80L, which carries a T5 IPTG‐inducible promoter (see Table S2 for oligonucleotide sequences and cloning restriction sites). All generated constructs used in this study are listed in Table S3.

**Expression of ncAA‐labeled target proteins with** ***MDS15*** **and** ***MDS15A***: Plasmids pSEVA26′*glnS*‐*metY‐metX* and pQE80L were introduced together into chemical competent *E. coli* expression strains *MDS15* and *MDS15A* by the heat shock method and plated on lysogeny broth (LB) agar with 100 μg/mL ampicillin and 50 μg/mL kanamycin. A single colony of transformed recombinant cells was selected and cultured in 5 mL LB medium with ampicillin and kanamycin at 37 °C and 200 rpm, overnight. The cells were pelleted for 10 min at 4 °C and 2000 g. The supernatant was carefully removed and the pellet gently resuspended in 2 mL of ENMM culture. The cells were inoculated into 1 L of ENMM medium with the required additional components, and the main culture was incubated at 37 °C, 200 rpm for 8 h for the first growth phase until the depletion of Met from the yeast extract. During this 8 h, 0.8 mM sodium azide and 1 mM pantothenic acid were added to the culture after 3, 41/2 and 6 hours. After this time, target protein expression was induced with 0.5 mM IPTG. The whole expression phase was continued overnight at 21 °C and 200 rpm. Each expression/labeling setup shown in Table 2 was repeated at least 3 times. The range of protein and label yield values was always less than ±5 %.

**Conjugation of oligoglycerol dendrimers on Aha‐labeled protein**: The click reaction was performed as previously described.^[48]^ Briefly, in a 500 μL reaction mixture was prepared as follows: Aha‐labeled protein (100 μL, 10 mg/mL in phosphate buffer (68 mM K_2_HPO_4_, 32 mM KH_2_PO_4_, 100 mM NaCl, pH 7.0), oligoglycerol dendrimer (dOG)[^65^, ^66^] (20 μL, 2 mM in H_2_O), 332.5 μL of phosphate buffer, aminoguanidine chloride (25 μL, 100 mM in H_2_O), l‐ascorbic acid (25 μL, 100 mM in H_2_O), copper‐mixture (7.5 μL, 20 mM copper(II) sulfate in H_2_O and 50 mM Tris(3‐hydroxypropyltriazolylmethyl)amine (THPTA) in H_2_O, 1 : 2 mixture). It was crucial that the THPTA and the l‐ascorbic acid solutions were freshly prepared. The reaction mixture was incubated at 4 °C overnight and then dialyzed against 1 L of phosphate buffer. Control conjugation experiments with unlabeled, Met‐containing target proteins, were performed and showed no oligoglycerol dendrimer conjugation.

### Purification of target proteins

*Purification of Barstar*: barstar protein was purified by ion‐exchange chromatography on a ÄKTA chromatography system. After induced expression, the cells were harvested by centrifugation for 10 min at 4000 g and 4 °C. The pellet was re‐suspended in 30 mL of 50 mM Tris ⋅ HCl, pH 8.0 and then 50 μL lysozyme were added and incubated for 30 min on ice. The mixture was then sonicated for 3 min on ice and centrifuged for 30 min at 3000 g and 4 °C. The supernatant was discarded, and the pellet was re‐suspended in the buffer with 50 mM Tris ⋅ HCl, pH 8.0 and 7.5 M urea. The mixture was then centrifuged for 30 min, at 15000 g and room temperature. The supernatant was extensively dialyzed (3500 Da cut‐off) against 50 mM Tris ⋅ HCl, pH 8.0, 100 mM NaCl. This was then centrifuged for 40 min at 22000 g and 4 °C, the lysate was passed through a 0.45 μm filter, and the entire sample was loaded into a superloop and purified with the 5 mL column of HiTrap Q‐sepharose with a linear elution gradient formed by 25 mL of equilibration buffer (50 mM Tris ⋅ HCl, pH 8.0, 100 mM NaCl) and 25 mL or elution buffer (50 mM Tris ⋅ HCl, pH 8.0, 1 M NaCl).

*Purification of His‐tagged target proteins*: His‐tagged target proteins were purified by Ni‐NTA chromatography. After cultivation, the cells were harvested and resuspended in 15 mL of loading buffer (50 mM Tris ⋅ HCl, 100 mM NaCl, 20 mM imidazole, pH 8.0). The cells were lysed by adding 0.1 mg/mL lysozyme, 0.1 mg/mL DNase and RNase, and the lysate was incubated at room temperature for 1 h. The cells were disrupted with a microfluidizer and centrifuged for 30 min at 22000 g and 4 °C. The supernatant was passed through a 0.45 μm filter. Chromatography was performed with peristaltic a pump P1 connected with a nickel HisTrap FF Crude column. The target proteins were eluted with a linear gradient formed by 25 mL of loading buffer and 25 mL of elution buffer. The eluate was then dialyzed against 5 L of TEV buffer (50 mM Tris ⋅ HCl, 100 mM NaCl, 0.5 mM EDTA, 1 mM DTT, pH 8.0) at 4 °C, overnight. The amount of TEV protease to be added was calculated by weight ratio 1 : 100 (*W*_tev_/*W*_GFP_). The digestion mixture was then incubated at room temperature, overnight. After TEV digestion, the protein was dialyzed against 5 L of the previously described Nickel Histrap loading buffer, overnight. Another Ni‐NTA chromatography was then performed in order to separate and remove the His_6_ tag fragment and the TEV protease from the purified target protein, which will not bind to the resin. The eluted proteins were then dialyzed against in storage buffer (50 mM Tris ⋅ HCl, 100 mM NaCl, 5 % glycerol, pH 8.0) at 4 °C, overnight, for the subsequent analysis or stored at −80 °C.

**ESI‐MS mass spectrometric analyses**: Mass spectra were collected on a 6500 Series accurate‐mass quadrupole time‐of‐flight (Q‐TOF) LC/MS (Agilent Technologies) connected with a C5 column. Data were managed by MassHunter data acquisition software provided with the instrument. Measurements were performed with buffer A (0.1 % formic acid in water) and buffer B (acetonitrile, 0.1 % formic acid), with a flow rate of 0.3 mL/min and injection volume of 10 μL sample. A measurement length of 33 min was employed, including 30 min linear change of buffer A from 95 to 40 %, buffer B from 5 to 60 % followed by 3 min of 100 % buffer B. Protein samples were diluted to 0.01–0.1 mg/mL with 1 % acetic acid/H_2_O prior to analysis. Data deconvolution analysis was performed with MassHunter Qualitative Analysis B.06.00 software integrated within the Agilent instrument.

***In vitro cg*** **OAHSS reaction test for diverse nucleophiles**: The *metX* gene encoding for *cg*HSAT was cloned into plasmid pQE80L with an N‐terminal His_6_ tag between SmaI and PstI restriction sites. The *metY* gene encoding for *cg*OAHSS was cloned to plasmid pBU26′*glnS* with a C‐terminal His_6_ tag between NheI and KasI restriction sites. The newly constructed plasmids pQE80L‐metX and pBU26′*glnS‐metY* were transformed to *E. coli* BL21(DE3) chemical competent cells by heat shock, separately. For *cg*HAST, the recombinant colony was cultured in 0.5 L LB medium with 100 μg/mL ampicillin at 37 °C, 200 rpm for 5 h, then induced by 1 mM IPTG, overnight. For the constitutively expressed *cg*OAHSS, a single colony of the recombinant *E. coli* strain was inoculated into 0.5 L LB medium with 50 μg/mL kanamycin and cultured at 37 °C and 200 rpm, overnight. Recombinantly expressed *cg*HAST and *cg*OAHSS proteins were purified by Nickel HisTrap FF crude column ÄKTA chromatography system with a linear elution gradient formed by 30 mL of equilibration buffer (50 mM Tris ⋅ HCl, pH 8.0, 100 mM NaCl, 20 mM imidazole) and 30 mL of elution buffer (50 mM Tris ⋅ HCl, pH 8.0, 1 M NaCl, 500 mM imidazole). The eluted proteins were dialyzed against reaction buffer (50 mM Tris ⋅ HCl, pH 8.0, 100 mM NaCl). For activity assays, the reaction mixture included the following components (with their final concentrations): l‐homoserine (10 mM), acetyl‐CoA (10 mM), diverse nucleophiles (20 mM), *cg*HSAT (5 μM), *cg*OAHSS (5 μM), in a total of 25 μL of reaction buffer. The reaction mixture was incubated at 37 °C for 1 h. After reaction, a 5 μL drop of mixture was loaded on thin layer chromatography (TLC pre‐coated aluminium silica gel plates, Sigma‐Aldrich). Chromatography was developed with *n*‐butanol/acetic acid/water (3 : 1 : 1, *v*/*v*/*v*) as eluent and stained by ethanolic ninhydrin (3 % *w/v*).

## Conflict of interest

The authors declare no conflict of interest.

## Supporting information

As a service to our authors and readers, this journal provides supporting information supplied by the authors. Such materials are peer reviewed and may be re‐organized for online delivery, but are not copy‐edited or typeset. Technical support issues arising from supporting information (other than missing files) should be addressed to the authors.

Supplementary
