# Supplementary material for: An Engineered Escherichia coli Strain with Synthetic Metabolism for in‐Cell Production of Translationally Active Methionine Derivatives
Source: Chembiochem. 2020 Oct 13;21(24):3525–38. doi: 10.1002/cbic.202000257 (PMC7756864; doi:10.1002/cbic.202000257)
Supplement: Supplementary file 1 — Supplementary [file CBIC-21-3525-s001.pdf]

ChemBioChem

Supporting Information

An Engineered *Escherichia coli* Strain with Synthetic Metabolism for in-Cell Production of Translationally Active Methionine Derivatives

Christian Johannes Schipp+, Ying Ma+, Ammar Al-Shameri, Federico D'Alessio, Peter Neubauer, Roberto Contestabile, Nediljko Budisa,* and Martino Luigi di Salvo*

Author Contributions

C.S. First author:Equal
Y.M. First author:Equal

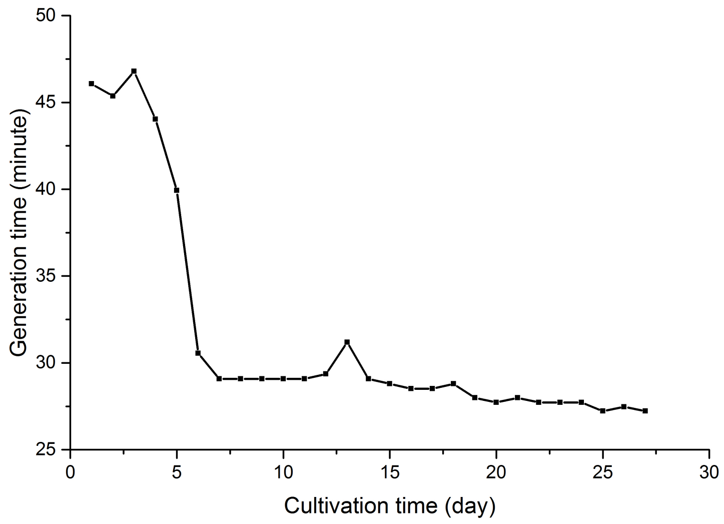

Figure S1. Direct evolution of *MDS15* strain in the Genemate3 turbidostat system. The generation time of the *E. coli* strain *MDS15* in the Genemate 3 turbidostat is calculated according to the dilution pulse frequency (see Materials and Methods section). Upon pressure of diluted medium, cells were evolved towards more robust growth and shorter generation time. The sharp peak near to the 13th day corresponds to routinary equipment adjustment in the Genemate 3 device. After 27 days of evolution, the generation time of MDS15 strain was decreased from 46 minutes to 27 minutes.

Barstar (B*)
MKKAVINGEQIRSISDLHQTLKKELALAEYYGENLDALWDALTGWV**M**YPLVLEWRQFEQSKQLTENGA
ESVLQVFREAKAEGADITIILS

GFP1M *Green fluorescent protein (1 Met residue)*
MRGSHHHHHHGSENLYFQSASKGEELFTGVVPILVELDGDVNGHKFSVRGEGEGDATNGKLTLKFICT
TGKLPVPWPTLVTTLGYGVQCFARYPDHIKRHDFFKSALPEGYVQERTISFKDDGTYKTRAEVKFEGD
TLVNRIELKGIDFKE**M**GNILGHKLEYNFNSHKVYITADKQKNGIKANFKIRHNVEDGSVQLADHYQQN
TPIGDGPVLLPDNHYLSTQSVLLKDPNEKRDHAVLLEFVTAAGITHGKDELYK

GFP2M *Green fluorescent protein (2 Met residue)*
MRGSHHHHHHGSENLYFQSASKGEELFTGVVPILVELDGDVNGHKFSVRGEGEGDATNGKLTLKFIC**M**
TGKLPVPWPTLVTTLGYGVQCFARYPDHIKRHDFFKSALPEGYVQERTISFKDDGTYKTRAEVKFEGD
TLVNRIELKGIDFKE**M**GNILGHKLEYNFNSHKVYITADKQKNGIKANFKIRHNVEDGSVQLADHYQQN
TPIGDGPVLLPDNHYLSTQSVLLKDPNEKRDHAVLLEFVTAAGITHGKDELYK

ECFP-N *Enhanced cyan fluorescent protein (His_6-Tag at the N-terminal)*
MRGSHHHHHHGS**M**VSKGEELFTGVVPILVELDGDVNGHKFSVSGEGEGDATYGKLTLKFICTTGKLPV
PWPTLVTTLTWGVQCFSRYPDH**M**KQHDFFKSA**M**PEGYVQERTIFFKDDGNYKTRAEVKFEGDTLVNRI
ELKGIDFKEDGNILGHKLEYNYISHNVYITADKQKNGIKANFKIRHNIEDGSVQLADHYQQNTPIGDG
PVLLPDNHYLSTQSALSKDPNEKRDH**M**VLLEFVTAAGITLG**M**DELYK

ECFP-C *Enhanced cyan fluorescent protein (His_6-Tag at the C-terminal)*
MVSKGEELFTGVVPILVELDGDVNGHKFSVSGEGEGDATYGKLTLKFICTTGKLPVPWPTLVTTLTWG
VQCFSRYPDH**M**KQHDFFKSA**M**PEGYVQERTIFFKDDGNYKTRAEVKFEGDTLVNRIELKGIDFKEDGN
ILGHKLEYNYISHNVYITADKQKNGIKANFKIRHNIEDGSVQLADHYQQNTPIGDGPVLLPDNHYLST
QSALSKDPNEKRDH**M**VLLEFVTAAGITLG**M**DELYKHHHHHH

GTL *Geobacillus thermocatenulatus lipase*
MRGSHHHHHHGSRENLYFQGAASRANDAPIVLLHGFTGWGREE**M**FGFKYWGGVRGDIEQWLNDNGYRT
YTLAVGPLSSNWDRACEAYAQLVGGTVDYGAAHAAKHGHARFGRTYPGLLPELKRGGRIHIIAHSQGG
QTAR**M**LVSLLENGSQEEREYAKAHNVSLSPLFEGGHHFVLSVTTIATPHDGTTLVN**M**VDFTDRFFDLQ
KAVLEAAAVASNVPYTSQVYDFKLDQWGLRRQPGESFDHYFERLKRSPVWTSTDTARYDLSVSGAEKL
NQWVQASPNTYYLSFATERTYRGALTGNYYPELG**M**NAFSAVVCAPFLGSYRNPTLGIDDRWLENDGIV
NTVS**M**NGPKRGSSDRIVPYDGALKKGVWND**M**GTYNVDHLEIIGVDPNPSFDIRAFYLRLAEQLASLRP

Figure S2. **Primary structure of target proteins used in this study**. Met residues are shown in red bold (**M**). His_6-Tag sequence is highlighted in cyan (HHHHHH); Tobacco Etch Virus endopeptidase (TEV) cleavage site is highlighted in green (EXLYΦQ\φ), being: X, any amino acid; Φ, large hydrophobic residue; φ, small hydrophobic/polar residue). TEV protease cuts after Q residue.

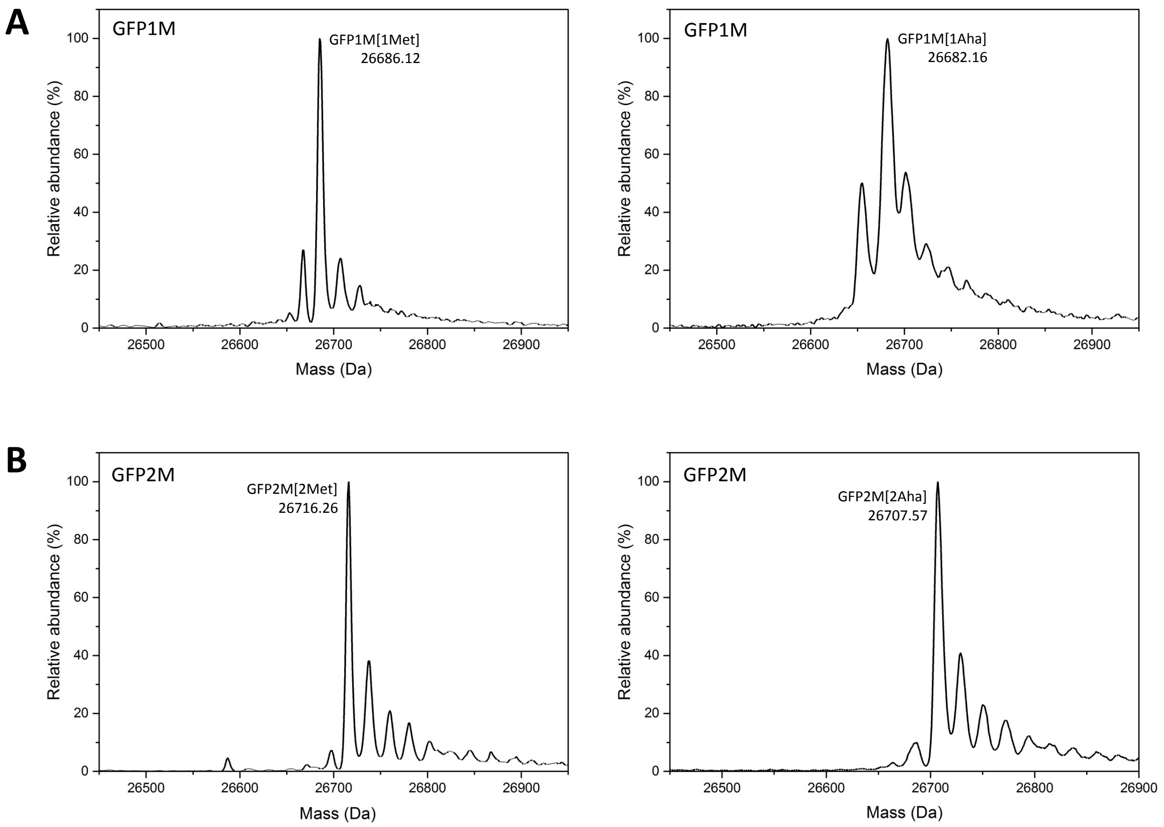

Figure S3. Mass spectrometry analysis of different variants of GFP. Panel A: GFP1M expressed in *MDS15A*, in the presence of Met (left) or according to our Aha production/incorporation system (right)(setup number 9 in Table 2); **Panel B: GFP2M** expressed in *MDS15A*, in the presence of Met (left) or according to our Aha production/incorporation system (right)(setup number 10 in Table 2). The substitution of each Met with Aha is expected to decrease the molecular weight of the protein by 5.1 Da. The values of deconvoluted and theoretical calculated masses are shown in Table 3. The additional signals around the main protein peaks represent salt adducts.

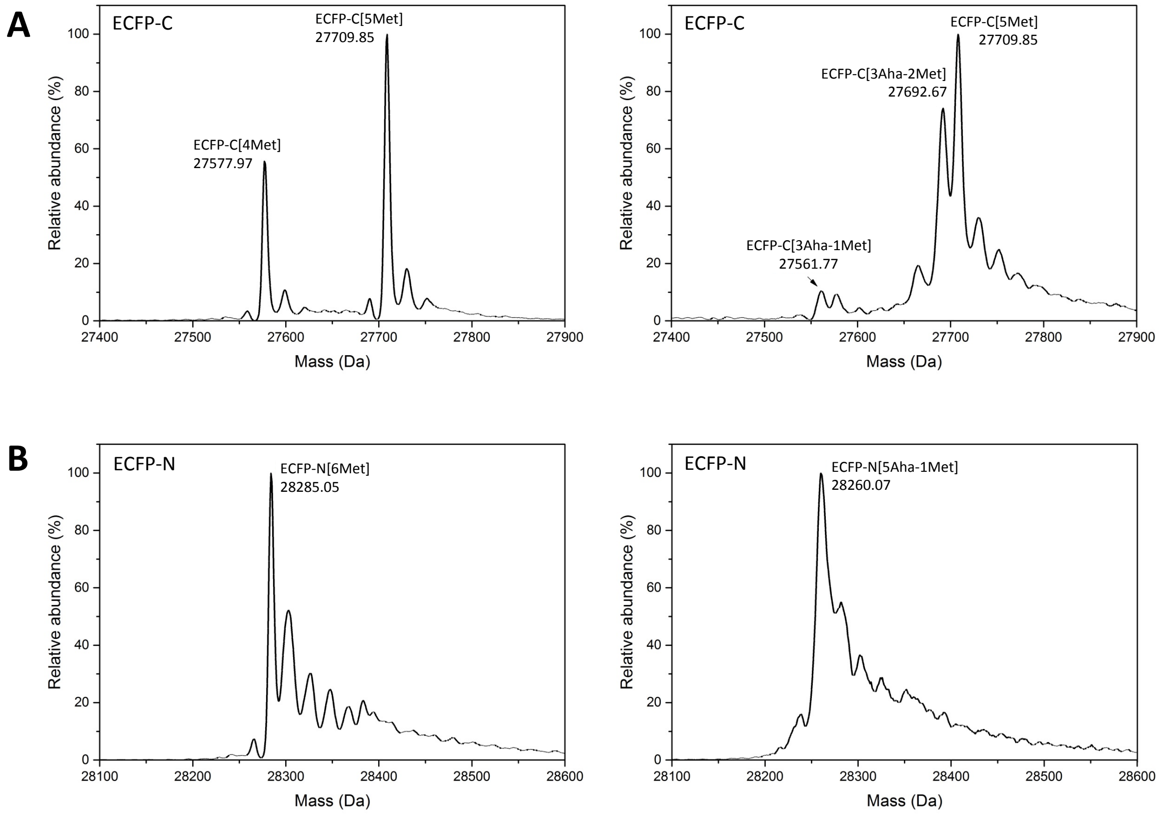

Figure S4. Mass spectrometry analysis of different variants of ECFP. Panel A: ECFP-C expressed in *MDS15A*, in the presence of methionine (left) or according to our Aha production/incorporation system (right)(setup number 5 in Table 2); **Panel B: ECFP-N** expressed in *MDS15A*, in the presence of methionine (left) or according to our Aha production/incorporation system (right)(setup number 8 in Table 2). The values of deconvoluted and theoretical calculated masses are shown in Table 3. The additional signals around the main protein peaks represent salt adducts.

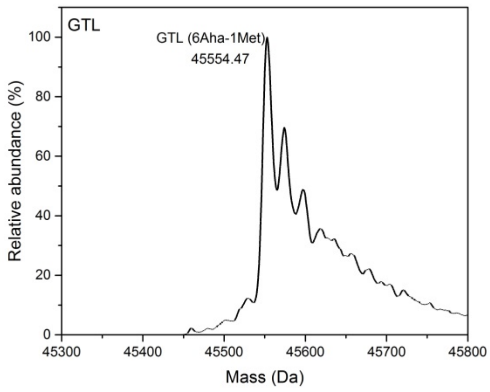

Figure S5. Mass spectrometry analysis of GTL, expressed in *MDS15* strain according to our Aha production/incorporation system (setup number 2 in Table 2).

As shown from the graphs, the deconvoluted mass was:
GTL[6Aha-1Met] 45554.47 Da

Theoretical masses calculated from primary structure is:
GTL[6Aha-1Met] 45554.65 Da

The additional signals around the main protein peak represent salt adducts.

Table S1. Bacterial strains used in this work (please, refer to Scheme 1 for metabolites and biosynthetic pathways reported in the **Description** column).

E. coli **strain**	**Genotype**	**Description**	**Source**
JW3973-1	*F⁻ Δ(araD-araB)567 ΔlacZ4787(::rrnB-3) λ- rph-1 Δ(rhaD-rhaB)568 ΔmetA780::kan hsdR514*	Knockout strain for *metA* (encoding endogenous homoserine *O*-succinyltransferase). Met-auxotroph. It is able to synthetize L-homoserine but not L-homocysteine. It can still synthetize L-methionine if fed with L-homocysteine.	CGSC-Keio collection
B834(DE3)	*F⁻ dcm ompT hsdS(rB- mB-) gal metλ (DE3 [lacI lacUV5-T7 gene 1 ind1 sam7 nin5]) Δmet E*	Knockout strain for *metE* (encoding endogenous methionine synthase). Met-auxotroph. This strain is still able to make L-homocysteine from L-homoserine, through the transsulfuration pathway.	Novagen (Merck-Millipore)
MDS15<br>*MDS15A*	*F⁻ dcm ompT hsdS(rB- mB-) gal metλ (DE3 [lacI lacUV5-T7 gene 1 ind1 sam7 nin5]) Δmet E ΔmetA780*	Double knockout strain, for *metA* and *metE*. Met-auxotroph. Not able to synthetize either L-homoserine or L-homocysteine. Not able to synthetize L-methionine even if fed with L-homocysteine.	This work
BL21-gold (DE3)	*B F- ompT hsdS(rB- mB-) dcm+ TetR gal λ (DE3) endA Hte*	Strain used to express the enzymes *cg*HSAT and *cg*OAHSS for *in vitro* test reaction	Novagen (Merck-Millipore)

Table S2. Primer list of oligonucleotides used in this study.

Primer name	Sequence	Note
metA-P1	5’−GCTATCTGGATGTCTAAACGTATAAGCGTATGTAGTGAGGTAATCAGGTTGTGTAGGCTGGAGCTGCTTC−3’	Forward primer for FRT-KanR-FRT cassette amplification for *metA* knockout
metA-P2	5’−GTGCCTGAGGTAAGGTGCTGAATCGCTTAACGATCGACTATCACAGAAGAATGGGAATTAGCCATGGTCC−3’	Reverse primer for FRT-KanR-FRT cassette amplification for *metA* knockout
glns-For	5’−CGCCCCTAGGCATCAATCATCCCCATAAT−3’	Forward primer for *glnS’-metY* cloning into pSEVA26’1, It carries *AvrII* restriction site (underlined).
metY-Rev	5’−AAAACCCGGGCTAGATTGCAGCAAAGCCGCC−3’	Reverse primer for *glnS’-metY* cloning into pSEVA26’1, It carries *SmaI* restriction site.
metX-For	5’−AAAACCCGGGTTTAACTTTAGAAGGAGGACAGCTATGCCCACCCTCGCGCCTTCAGGTCAA−3’	Forward primer for *metX* cloning into pSEVA26’1, It carries *SmaI* restriction site.
metX-Rev	5’−CCGCTCTAGATTAGATGTAGAACTCGATGTAGGTCGAAGGGTTGTCTTCGTC−3’	Reverse primer for *metX* cloning into pSEVA26’1, It carries *SmaI* restriction site.
metY-his-For	5’−CGCCGCTAGCCCAAAGTACGACAATTCCA−3’	Forward primer for *metY* cloning into pBU26’*glnS*, It carries *Nhe*I restriction site.
metY-his-Rev	5’−AAAAGGCGCCCTAGTGATGGTGATGGTGATGGATTGCAGCAAAGCCGCC−3’	Reverse primer for *metY* cloning into pBU26’glnS, It carries *Kas*I restriction site.
metX-his-For	5’−AAAACCCGGGATGCCCACCCTCGCGCCTTCAGGTCAA−3’	Forward primer for *metX* cloning into pQE80L, It carries *Sma*I restriction site.
metX-his-Rev	5’−CCGCCTGCAGCTTAGATGTAGAACTCGATGTAGGTCGAAGGGTTGTCTTCGTC−3’	Reverse primer for *metX* cloning into pQE80L, It carries *PstI* restriction site.
B*-For	5’−AAAAGAATTCGAGCTCTAGAGTCCGGTC−3’	Forward primer for inserting cDNA encoding barstar (2 Met version) into pQE80L. It carries *EcoR*I restriction site.
B*-Rev	5’−AAGCTTGCGGGTTTGTGTTTCCATA−3’	Reverse primer for inserting cDNA encoding barstar (2 Met version) into pQE80L. It carries *Hind*III restriction site.
GTL-For	5’−CCGCGGATCCAGAGAAAACTTGTATTTC−3’	Forward primer for inserting cDNA encoding GTL into pQE80L. It carries *BamH*I restriction site.
GTL-Rev	5’−AAAACTGCAGTTATTAAGGCCGCAAACT−3’	Reverse primer for inserting cDNA encoding GTL into pQE80L. It carries *Pst*I restriction site.
ECFP-N-For	5’−AAAAGGATCCATGGTGAGCAAGG−3’	Forward primer for inserting cDNA encoding ECFP-N into pQE80L. It carries *BamH*I restriction site.
ECFP-N-Rev	5’−AAGCTTTTATCACTTGTACAGCTCG−3’	Reverse primer for inserting cDNA encoding GTL into pQE80L. It carries *Hind*III restriction site.
ECFP-C-For	5’−CCGCGAATTCATTAAAGAGGAGAAATTAACTATGGTGAGC−3’	Forward primer for inserting cDNA encoding ECFP-C into pQE80L. It carries *EcoR*I restriction site.
ECFP-C-Rev	5’−AAGCTTAGTGATGGTGATGGTGATGC−3’	Reverse primer for inserting cDNA encoding ECFP-C into pQE80L. It carries *Hind*III restriction site.
GFP−For	5’−AAAAGGATCCGAGGCCTGTACTTCCAATCCGCGA−3’	Forward primer for inserting cDNA encoding for GFP (both 1M and 2M version) into pQE80L. It carries *BamH*I restriction site.
GFP−Rev	5’−CGCCAAGCTTTCATTTATACAGTTCATCTTTGCCG−3’	Reverse primer for inserting cDNA encoding for GFP (both 1M and 2M version) into pQE80L. It carries *Hind*III restriction site.

Table S3. Plasmidic constructs used in this study.

Construct	Origin of replication	Resistance	Description
pSEVA26'*glnS-metY-metX*	P15A	Kan	Construct constitutively expressing *cg*HSAT and *cg*OAHSS for in-cell production of Aha in *MDS15* and *MDS15A E. coli* strains.
pQE80L-*metX*	ColE1	Amp	Construct expressing *cg*HSAT with N-terminal HisTag for *in vitro* test.
pBU26'*glnS-metY*-his	P15A	Kan	Construct constitutively expressing *cg*OAHSS with C-terminal His Tag for *in vitro* test.
pQE80L-B*	ColE1	Amp	Construct expressing B* under IPTG induction
pQE80L-ECFP-N	ColE1	Amp	Construct expressing enhanced cyan fluorescent protein with N-terminal His_6-tag, under IPTG induction
pQE80L-ECFP-C	ColE1	Amp	Construct expressing enhanced cyan fluorescent protein with C-terminal His_6-tag, under IPTG induction
pQE80L-GTL	ColE1	Amp	Construct expressing *Geobacillus thermocatenulatus* lipase, under IPTG induction
pQE80L-GFP-1M	ColE1	Amp	Construct expressing green fluorescent protein from *Aequorea victoria* (carrying one methionine residue), under IPTG induction.
pQE80L-GFP-2M	ColE1	Amp	Construct expressing green fluorescent protein from *Aequorea victoria* (carrying two methionine residue), under IPTG induction.
pKD46	oriR101; w/repA101ts	Amp	For making chromosomal deletions of genes with FRT sites. Temperature sensitive replication; encodes lambda Red genes (*exo*, *bet*, *gam*); arabinose-inducible promoter.
pKD4	oriR6Kgamma	Kan	Template plasmid for FRT-flanked kanamycin cassette
pCP20	oriR101	Amp	Temperature-sensitive origin of replication; encodes the FLP recombinase. Used to eliminate kanamycin resistance cassette from *E. coli* strains.

Table S4. Compounds tested *in vitro* as nucleophiles for *cg*OAHSS reaction. The plus (+) sign in the **Reactivity** column denotes that the compound was successfully condensed with L-homoserine in the presence of the enzyme. Product formation was detected by TLC chromatography and ninhydrin stain (data not shown). The minus (-) sign denotes that no reaction occurred under our experimental conditions.

Name	Structure	Reactivity	Name	Structure	Reactivity
Pyrrole	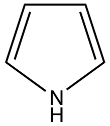	-	1,2,3,4-Tetrazole	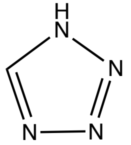	-
Indole-3-acetic acid	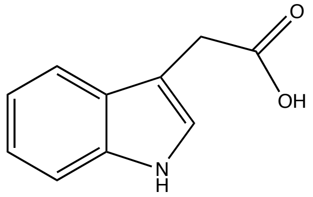	-	6-Benzylaminopurine	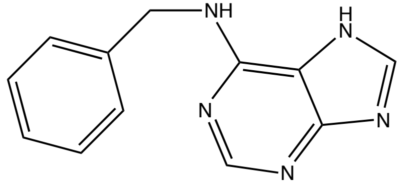	-
2-Aminoisobutyric acid	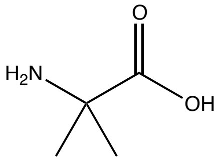	-	Sodium sulphide	S_2^-	+
1,2 Pyrazole	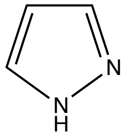	+	Sodium thiosulfate	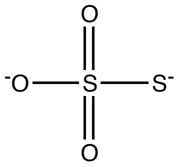	+
Imidazole	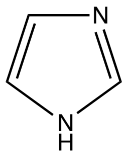	-	2-Mercaptoethanol	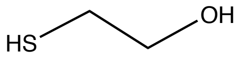	+
Pyridazine	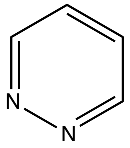	-	2-Mercaptoethylamine	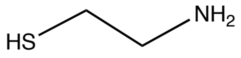	+

Phenylhydrazine	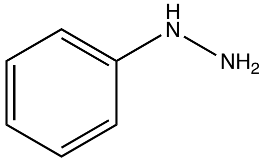	-	L-Cysteine	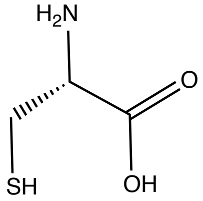	-
Sodium azide	$^{-}N{=}N^{+}{=}N^{-}$	+	Hypotaurine	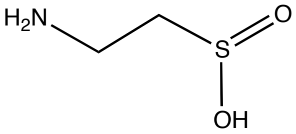	-
1,2,3-Benzotriazole	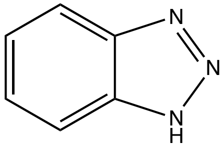	+	Benzenethiol (Thiophenol)	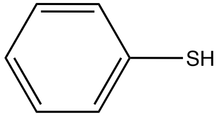	+
1,3-Benzodioxole	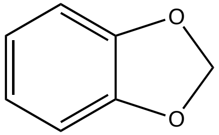	+	Benzeneselenol (Phenylselenol)	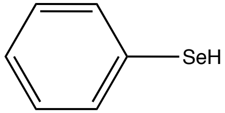	+
5-Carboxy-1,2,3-Benzotriazole	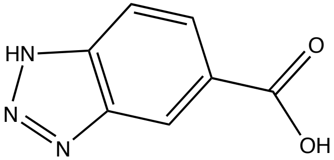	-	Benzyl mercaptan	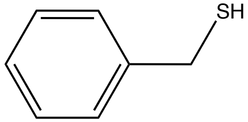	+
1,2,4-Triazole	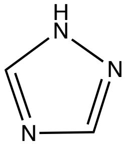	-	2-Propene-1-thiol (Allyl mercaptan)	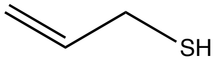	+
